# Geospatial distributions reflect temperatures of linguistic features

**DOI:** 10.1126/sciadv.abe6540

**Published:** 2021-01-01

**Authors:** Henri Kauhanen, Deepthi Gopal, Tobias Galla, Ricardo Bermúdez-Otero

**Affiliations:** 1 Zukunftskolleg, University of Konstanz, Universitätsstraße 10, 78464 Konstanz, Germany.; 2 Department of Theoretical and Applied Linguistics, University of Cambridge, Sidgwick Avenue, Cambridge CB3 9DA, UK.; 3 Department of Physics and Astronomy, School of Natural Sciences, The University of Manchester, Oxford Road, Manchester M13 9PL, UK.; 4 Instituto de Física Interdisciplinar y Sistemas Complejos (IFISC), CSIC-UIB, Campus Universitat Illes Balears, E-07122 Palma de Mallorca, Spain.; 5 Department of Linguistics and English Language, The University of Manchester, Oxford Road, Manchester M13 9PL, UK.

## Abstract

Quantifying the speed of linguistic change is challenging because the historical evolution of languages is sparsely documented. Consequently, traditional methods rely on phylogenetic reconstruction. Here, we propose a model-based approach to the problem through the analysis of language change as a stochastic process combining vertical descent, spatial interactions, and mutations in both dimensions. A notion of linguistic temperature emerges naturally from this analysis as a dimensionless measure of the propensity of a linguistic feature to undergo change. We demonstrate how temperatures of linguistic features can be inferred from their present-day geospatial distributions, without recourse to information about their phylogenies. Thus, the evolutionary dynamics of language, operating across thousands of years, leave a measurable geospatial signature. This signature licenses inferences about the historical evolution of languages even in the absence of longitudinal data.

## Teaser

The geographical distributions of features of language contain a footprint of the cultural evolutionary dynamics of language.

## INTRODUCTION

Since the biological emergence of modern language some 100,000 years ago (*1*), human languages have diversified through processes of cultural evolution to the extent that thousands of distinct languages are spoken today around the world (*2*). These languages display an enormous amount of variation in a combinatorial space spanned by a finite number of structural features, whose possible values emerge from biological and cognitive constraints on linguistic representation and language use. These features determine how individual words are formed, how words are combined into phrases and sentences, and which sounds and sound sequences are available in any given language.

The causes of linguistic change have been debated ever since the birth of modern linguistic theory in the late 19th century, and a number of these processes are now understood in detail (*3*). The most basic general insight emerging from this work, translated into terms that are current in the study of evolution in other fields (*4*), is that language change is both vertical and horizontal. Under ordinary circumstances, language is relatively reliably passed on from parents to children, which accounts for the vertical, intergenerational descent of linguistic features across phylogenetic lineages. It is possible for this transmission to fail, however, and for a feature to change, a process not unlike point mutations in the genome, although debate exists over whether linguistic mutations are mostly random or directed (*5*). It is also possible for the vertical line of descent to be interrupted by horizontal effects, in which a feature of a language changes because of the influence of a phylogenetically distinct but geographically neighboring language; the empirical problem of distinguishing the results of horizontal effects from the results of failed vertical transmission recurs often in many areas of historical linguistics (*3*).

Within the study of the dynamics of language, there is a large and rich body of work that seeks to measure the susceptibility of linguistic features to change over time (*6*–*15*). In this tradition, susceptibility to change is evaluated in terms of linguistic stability, which is generally understood as resistance to endogenous change, that is, resistance to mutation in vertical transmission, to the exclusion of horizontal effects. Consider two protolanguages *L* and *L*′ at a given point in historical time, such that *L* has feature *F*, while *L*′ lacks this feature. After a suitable period of time, if all the descendants of *L* have feature *F* and all the descendants of *L*′ lack it, then *F* is said to display maximal stability over this time period. Conversely, *F* is said to display maximal instability over this time period if it is found that any individual descendant of *L*′ has exactly the same probability of having feature *F* as any individual descendant of *L*. This ideal scenario assumes that the only forces acting on *L*, *L*′, and their descendants pertain to intergenerational transmission so that there are no horizontal effects of language contact.

In this light, the tradition of linguistic research described above sees it as a key task to devise methods of stability estimation that can effectively control for the role of horizontal contact in the evolutionary dynamics of language, recovering the vertical signal as cleanly as possible. Some approaches within this tradition rely solely on phylogenetic information, i.e., information about the distribution of linguistic features among groups of related and unrelated languages (*7*, *11*), while others combine phylogenetic and areal information (*8*, *9*, *15*). In general, however, these approaches seek to control for horizontal effects in an effort to isolate stability in the vertical dimension. For convenience, therefore, we may refer to this tradition as “the stability program” or “the vertical program.”

One complication facing the vertical program is that the actual dynamics of the cultural evolution of language do exhibit extensive horizontal effects; idealized vertical stability is not always recoverable from the signal. Attested situations of horizontal transfer, in which features are borrowed from one language family to another, range from the multilingualism and diglossia that characterize the linguistic landscapes of major cosmopolitan centers to more intricate situations of language contact between two or more geographically contiguous language communities (*16*). The problem for phylogenetic stability estimation methods arises in these situations from the fact that some linguistic features (e.g., inflectional markers) are known to be more resistant to horizontal transfer than others (*17*), while some (e.g., case systems) are highly vulnerable to simplification in contact situations involving large numbers of second-language (L2) learners (*18*): There is a complex dependence of the rate of horizontally motivated change on both the type of contact situation and the nature of the feature itself. Enriching the phylogenetic analysis with areally defined groupings (*8*, *15*) is only a partial solution, however, as no agreed methods exist for delimiting linguistic areas.

A related contrast exists in the field of population genetics between phylogeographic approaches and methods that rely on mathematical models and summary statistics (key quantities summarizing an observation) to infer properties of genetic evolution (*19*). Noting this fact, here, we pursue a model-based approach to the dynamics of language change. We treat the cultural evolution of language as a combination of two stochastic processes, one operating in the vertical domain and the other operating in the horizontal domain. From this model, we derive a quantity, which we call (linguistic) temperature, which expresses the global ratio of unfaithful transmission in both the vertical and horizontal dimensions (mutation) to faithful transmission. By this definition, temperature is different from stability, as defined within the vertical program. Nonetheless, we expect, and indeed demonstrate below, that temperature estimates will be strongly correlated with the stability estimates generated by phylogenetic methods. The expectation of these correlations builds on conceptual considerations, which suggest that the mechanisms of endogenous language change (i.e., the mechanisms that cause mutations in the vertical dimension of intergenerational transmission) are, in fact, not entirely independent from the mechanisms of contact-driven language change (i.e., the mechanisms that cause mutations in the horizontal dimension of language contact).

As we show in detail below, the temperature of a linguistic feature can be recovered using this model if two empirically measurable “summary statistics” about that feature are known, the feature’s overall frequency across a sample of languages and a measure of how clumped or scattered the feature is in geographical space. The consequence is that linguistic temperature, a dimensionless measure of the propensity of a linguistic feature to undergo change, is recoverable from synchronic information about that feature’s empirical geospatial distributions, without recourse to information about its phylogeny.

## RESULTS

### Modeling the stochastic dynamics of language

The transmission of a linguistic feature can be faithful or unfaithful whether it takes place on the vertical dimension (i.e., intergenerationally) or on the horizontal dimension (i.e., through language contact). As above, faithful transmission in the vertical dimension results in historical stability (in the technical sense of the existing literature), whereas unfaithful transmission in the vertical dimension amounts to endogenous change. On the horizontal dimension, however, transmission can also be either faithful or unfaithful. Faithful horizontal transmission results in simple transfer between languages by contact, often called borrowing (*16*). In contrast, unfaithful horizontal transmission occurs when adult learners of an L2 incorporate into their first language (L1) a modified, typically simplified, version of a feature of L2. This sort of simplification by L2 learners is widely thought to underlie phenomena such as the emergence of impoverished inflectional systems from contact between languages with rich but heterogeneous inflections (*18*).

**Fig. 1:**
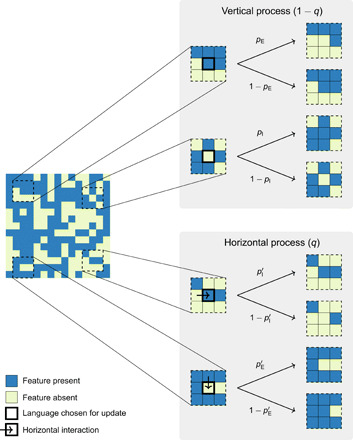
Illustration of the model dynamics. At each iteration, a random cell of the lattice is chosen for update. A randomly selected feature then undergoes either a horizontal event (with probability *q*) or a vertical event (with probability 1 *− q*). The value of the feature may flip (from “absent” to “present” or vice versa) because of ingress or egress in either type of event. In a horizontal event, the donor language is drawn randomly from among the focal language’s four von Neumann neighbors.

To model these interactions, we expand upon an early but underexploited paradigm in dynamic linguistic typology, which proposed to model the dynamics of language as a Markov process in the vertical domain (*20*). This work also suggested, albeit without putting forward a mathematically explicit model of spatial diffusion, that features attesting different ingress and egress rates ought to pattern differently geographically. We here make these assumptions concrete by implementing languages on a spatial substrate; a similar approach, based on computer simulations of a more complex dynamics, has been pursued in (*9*). For the sake of mathematical tractability, we assume languages to be distributed on a regular square lattice and each feature to be binary (absent or present) in any given language. Each language on the lattice is subject to a vertical and a horizontal process with respect to each of its features; in our stylized model, we assume that each feature evolves independently of the other features. The model has five free parameters per feature, *p*_I_, *p*_E_, $p_{I}^{\prime }$, $p_{E}^{\prime }$, and *q*, each a probability. In the vertical process, transmission errors occur at rates *p*_I_ and *p*_E_, where *p*_I_ is the probability of innovating a feature that the language lacks (called the feature’s vertical ingress rate) and *p*_E_ is the probability of losing an existing feature (called the feature’s vertical egress rate). In the horizontal process, a feature (or its absence) is copied into the language from one of its immediate neighbors on the lattice. This horizontal transfer is subject to errors as follows: A feature’s absence is incorrectly copied as its presence with probability $p_{I}^{\prime }$ (horizontal ingress rate), while its presence is incorrectly copied as its absence with probability $p_{E}^{\prime }$ (horizontal egress rate). A fifth parameter, *q*, supplies the relative rate of horizontal over vertical events (see Fig. 1 for a summary illustration and Materials and Methods for a complete algorithmic specification of the model).

Similar building blocks (copying, diffusion, and mutation) have been used in other fields, for example, in modeling the dynamics of opinions in the social sciences (*21*). Notably, in biology, models of this general type are often a key component of genetic inference based on summary statistics (*19*). So-called stepping stone, island, or voter models involve combinations of copying processes and mutation (*21*–*24*), with some differences in the details of the implementation of these components. In some models, each spatial node is occupied by multiple agents (*22*), while in others, each node hosts one agent only, similar to our model setup (*23*). The model we use can perhaps best be described as an “asymmetric noisy two-state voter model” (*25*). Its behavior, similar to that of related models, can be studied using methods and concepts from statistical physics (*21*, *26*–*28*). In particular, key quantities describing the stationary state can be computed. In our case, these are properties of the distribution of features across the lattice. Our approach is therefore similar in spirit to work in population genetics focusing on the inference of parameters of evolutionary processes from summary statistics of observed patterns of genetic diversity, using analytical solutions of stylized models of evolution (for example, via coalescent theory) (*19*, *28*–*31*).

**Fig. 2:**
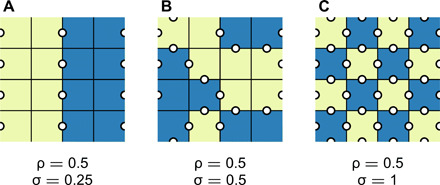
Feature frequency and isogloss density. In each of these schematic illustrations, the feature frequency is ρ = 0.5 (half of the sites are yellow, the other half are blue). However, isogloss density σ, defined as the proportion of disagreeing lattice interfaces (white dots), depends on the spatial distribution of the feature. It is low when a feature is present throughout extended domains (**A**), intermediate when a feature is randomly distributed (**B**), and large when a feature is scattered (**C**).

In our lattice model, the statistical properties of the stationary distribution of a linguistic feature depend on the feature’s parameters *p*_I_, *p*_E_, $p_{I}^{\prime }$, $p_{E}^{\prime }$, and *q*. Useful information about the stationary distribution is contained in two quantities, illustrated in Fig. 2: the frequency ρ with which a particular feature is present across the lattice and the feature’s associated isogloss density σ. The latter quantity is defined as the probability of finding a dialect boundary (an isogloss) between two neighboring languages such that the feature is found on one side of the boundary but not on the other; similar quantities are sometimes also found as the “density of active interfaces” or “active bonds” [see (*21*) and references therein]. Our model is more stylized than that in (*9*), for instance, and as a consequence of this austere setup, values of ρ and σ in the stationary distribution can be calculated analytically in the special case $p_{I}^{\prime }=p_{E}^{\prime }$, i.e., when errors in the horizontal transmission of language are not directed. This calculation follows well-established principles in statistical physics (*21*, *27*), particularly the procedure in (*26*). The following results are a generalization of the analytical solution, verified in numerical simulations for the general case in which $p_{I}^{\prime }$ and $p_{E}^{\prime }$ are independent. We refer the reader to the Supplementary Materials for the analytical derivations and a full description of the numerical simulations.

The frequency of a feature in the stationary distribution is given by

$$\rho =\frac{(1-q)p_{I}+qp_{I}^{\prime }}{(1-q)p+qp^{\prime }}$$, (1)

where *p* = *p*_I_ + *p*_E_ and $p^{\prime }=p_{I}^{\prime }+p_{E}^{\prime }$ represent the total error rates of the vertical and horizontal processes, respectively. For the isogloss density, we find

σ=2H(τ)ρ(1−ρ) (2)

with

$$H(\tau )=\frac{\pi (1+\tau )}{2K(\frac{1}{1+\tau })}-\tau$$ (3)

and

$$\tau =\frac{(1-q)p+qp^{\prime }}{q(1-p^{\prime })}$$. (4)

**Fig. 3:**
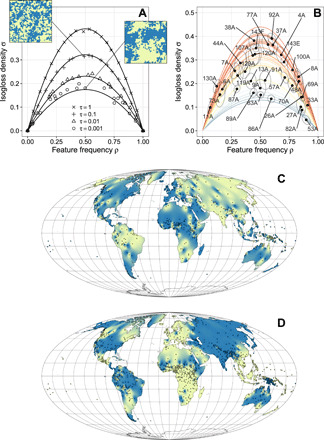
Statistical properties of the model and empirical measures of feature frequency and isogloss density. (**A**) At long times, the state of the lattice is characterized by the two quantities feature frequency ρ and isogloss density σ. We show computer simulations (markers) and analytical solution (curves) for different values of τ. Simulation snapshots of the lattice are shown for two different values of τ. (**B**) Empirical measurements of feature frequency ρ and isogloss density σ for 35 linguistic features, identified by their World Atlas of Language Structures (WALS) feature IDs (see Table 1 for a key), with 95% confidence ellipses from the empirical bootstrap. (**C**) Empirical geospatial distribution of WALS feature 37A (definite marker). (**D**) Empirical geospatial distribution of WALS feature 83A (OV word order). Shown are both individual empirical data points (languages, as given by WALS coordinates) and a spatial interpolation (inverse distance weighting) on these points. Blue, feature present; yellow, feature absent. Map projection, Mollweide equal area.

The function *K*(·) denotes the complete elliptic integral of the first kind. From Eq. 2, the stationary-state isogloss density σ is found to be a parabolic function of the feature’s overall frequency ρ. The height of this parabola is controlled by *H*(τ) and, hence ultimately, by the parameter τ (Fig. 3A). This parameter gives the relative rate of unfaithful transmission events (i.e., mutations) over faithful transmission events (Eq. 4) and can thus be interpreted as a dimensionless measure of the propensity of the feature to undergo change: Lower values of τ signify a relatively infrequently changing feature, while higher values indicate relative rapidity in change. (In Eq. 4, the denominator does not include a term for faithful transmission events in the vertical process. This may be puzzling at first but becomes more natural when one realizes that faithful vertical events never change the state of the lattice. These events promote neither order nor disorder, and temperature as an overall measure of disorder is hence not affected by the background of faithful vertical transmission.) We refer to τ as temperature, and note that, as a dimensionless ratio, it is not calibrated to an overall frequency of transmission events in language. Such a calibration is unnecessary for our purposes, as we are only interested in the relative ranking of different features in terms of τ.

### Inferring linguistic temperatures from geospatial distributions

To arrive at empirical estimates of temperatures of linguistic features, we then need data from which feature frequencies (ρ) and isogloss densities (σ) can be measured. These data are available in the World Atlas of Language Structures (WALS) (*32*), a large-scale typological database also containing spatial information in the form of geographical coordinates for languages. We curated 35 binary or binarized features from the WALS, each of which is recorded for at least 300 languages in the atlas (for full details, see Materials and Methods). For each feature, the frequency ρ is given by the proportion of languages in which that feature is present (rather than absent) in the feature’s WALS language sample. Isogloss density σ was estimated using the 10 geographically nearest-neighbor languages of each language in the sample. Last, the analysis was repeated 1000 times, resampling languages with replacement to generate bootstrap confidence intervals. The results are summarized in Fig. 3B, which supplies median feature frequency ρ and isogloss density σ for each of the 35 features, together with 95% bootstrap confidence ellipses. Figure 3 (C and D) provides a detailed illustration of the geospatial distribution of two features, definite marker (WALS feature 37A), and order of object and verb (WALS feature 83A).

**Fig. 4:**
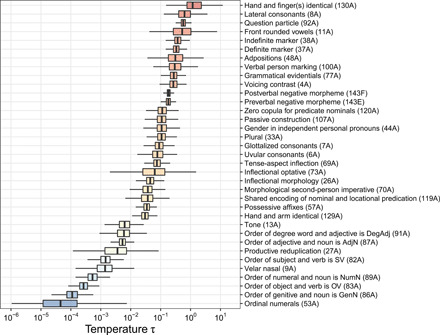
Temperature estimates for the 35 WALS features considered in this study. The box plots show the bootstrap distributions over 1000 runs; central bars represent medians.

For a given feature frequency ρ, the isogloss density σ is fixed by the value of *H*(τ) (Eq. 2); this quantity itself is an increasing function of τ (Eq. 3). Since each of our 35 empirical features lies on a unique parabola in the space spanned by ρ and σ (Fig. 3), estimating its temperature is now a matter of inverting the function *H*(τ). Although the elliptic integral in Eq. 3 cannot be expressed in terms of elementary functions and *H*(τ) thus cannot be inverted analytically, the inversion can be performed numerically (see Materials and Methods). Using this procedure, we obtain an estimate of τ for any feature for which empirical measurements of frequency ρ and isogloss density σ exist. Figure 4 supplies the bootstrap distributions of these estimates (for numerical values of the medians, see Table 1). Estimated temperatures span a range of roughly five orders of magnitude, with word order features tending to have the lowest temperatures and certain lexical, phonological, and morphological features the highest.

**Table 1:** The 35 linguistic features consulted in this study, ranked in order of decreasing estimated temperature. ρ, frequency of feature; σ, isogloss density; τ, temperature; median values across 1000 bootstrap samples.

**Rank**	**Feature**	**WALS ID**	**ρ**	**σ**	**τ**
1	Hand and finger(s) identical	130A	0.12142	0.18876	1.21789
2	Lateral consonants	8A	0.83245	0.22907	0.63618
3	Question particle	92A	0.59955	0.39077	0.56961
4	Front rounded vowels	11A	0.06584	0.09890	0.53281
5	Indefinite marker	38A	0.44569	0.38193	0.37116
6	Definite marker	37A	0.60806	0.36313	0.33385
7	Adpositions	48A	0.83333	0.20930	0.31049
8	Verbal person marking	100A	0.77895	0.25744	0.29686
9	Grammatical evidentials	77A	0.56699	0.36507	0.27513
10	Voicing contrast	4A	0.68078	0.32122	0.26579
11	Postverbal negative morpheme	143F	0.46224	0.34988	0.18558
12	Preverbal negative morpheme	143E	0.70544	0.29141	0.18094
13	Zero copula for predicate nominals	120A	0.45337	0.32420	0.11337
14	Passive construction	107A	0.43432	0.31892	0.10915
15	Gender in independent personal pronouns	44A	0.32804	0.28686	0.10814
16	Plural	33A	0.90901	0.10821	0.10752
17	Glottalized consonants	7A	0.27866	0.25397	0.08912
18	Uvular consonants	6A	0.17108	0.17617	0.07762
19	Tense-aspect inflection	69A	0.86561	0.14372	0.07507
20	Inflectional optative	73A	0.15047	0.15337	0.06375
21	Inflectional morphology	26A	0.85552	0.14203	0.04492
22	Morphological second- person imperative	70A	0.77697	0.19176	0.03745
23	Shared encoding of nominal and locational predication	119A	0.30311	0.23385	0.03724
24	Possessive affixes	57A	0.71175	0.22579	0.03463
25	Hand and arm identical	129A	0.36791	0.25061	0.03016
26	Tone	13A	0.41746	0.21060	0.00626
27	Order of degree word and adjective is DegAdj	91A	0.54177	0.21376	0.00595
28	Order of adjective and noun is AdjN	87A	0.29736	0.17745	0.00533
29	Productive reduplication	27A	0.85054	0.10143	0.00361
30	Order of subject and verb is SV	82A	0.86013	0.08752	0.00145
31	Velar nasal	9A	0.49893	0.18057	0.00139
32	Order of numeral and noun is NumN	89A	0.44015	0.16075	0.00053
33	Order of object and verb is OV	83A	0.50317	0.15203	0.00027
34	Order of genitive and noun is GenN	86A	0.59497	0.13545	0.00011
35	Ordinal numerals	53A	0.89720	0.04624	0.00005

### Comparison with a phylogenetic method

We have predicted on conceptual grounds that our estimates of linguistic temperature (τ) will be correlated with estimates of vertical stability. To test this prediction, we choose a method of vertical stability estimation that is diametrically opposed to our own spatial procedure of temperature estimation: namely, a method that operates on phylogenetic data to the exclusion of any spatial signal. Dediu (*11*) recovered estimates of the rate of evolution of a selection of linguistic features using two different Bayesian phylogenetic methods and drawing data from two sources, WALS and Ethnologue (*33*), to control for possible implementation effects. The aggregate rate estimates from this analysis are expressed as the additive inverse of the first component of a principal components analysis (PC1) on the evolution rate rank predicted by each combination of phylogenetic algorithm and dataset. In practice, the higher the PC1 value, the higher the evolution rate of the feature and, consequently, the lower its stability.

**Fig. 5:**
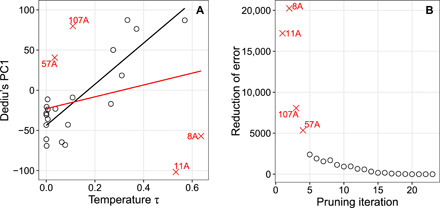
Regression of our temperature estimates (τ) against Dediu’s PC1 (*11*). (**A**) Red line indicates regression with all 24 data points (Spearman’s *r*_S_ = 0.37, *P* = 0.08). Black line indicates regression with four outliers (red crosses) removed (Spearman’s *r*_S_ = 0.68, *P* < 0.01). (**B**) Outliers were detected by pruning the dataset recursively for those data points that contributed most to the regression error, quantified as the sum of squared residuals. This identified features 11A, 8A, 107A, and 57A as outliers (see main text).

In Fig. 5, we consider the 24 features that fall in the intersection of our list of 35 features and Dediu’s list. Regressing our (median) estimates for τ against Dediu’s PC1 (red regression line), we find no evidence of a correlation between the estimates predicted by the two methods (Spearman’s *r*_S_ = 0.37, *P* = 0.08). A number of features, however, are clearly outliers of the regression. To detect these outliers more objectively, we pruned the regression recursively by removing those data points that contributed the greatest error in terms of sum of squared residuals. This procedure classified as outliers the following WALS features: 11A (front rounded vowels), 8A (lateral consonants), 107A (passive construction), and 57A (possessive affixes). Regressing the pruned dataset (Fig. 5, black regression line), we find a significant high correlation between our τ estimates and Dediu’s PC1 (Spearman’s *r*_S_ = 0.68, *P* < 0.01).

We suggest that, rather than instantiating a lack of correlation between stability (as understood in the vertical program) and temperature, these outliers are false positives and false negatives of the purely phylogenetic method of stability estimation in (*11*). This is illustrated by the case of front rounded vowels (WALS feature 11A), i.e., the presence or absence of the vowels /y/ (e.g., Finnish *kyy*), /ø/ (German *schön*), /œ/ (French *bœuf*), and /Œ/ (Danish *grøn*) in a language’s phonology (*13*). Front rounded vowels are one of the most stable features in the genetic analysis [they are the fourth most stable feature (out of 86) in Dediu’s study (*11*) and the second most stable (out of 62) in the meta-analysis in (*12*)] but one of the highest-temperature features in ours (Fig. 4). We argue that evidence from both language change and language acquisition supports our position. On the one hand, front rounded vowels are frequently innovated: historical fronting of the back rounded vowel /u/ to [y] (with or without subsequent phonemicization to /y/) has been documented in a number of languages (*34*). In addition, front rounded vowels can arise through the influence of /i/ or /j/ on a neighboring back rounded vowel (*35*). On the other hand, front rounded vowels are difficult to acquire in situations of language contact: Laboratory experiments have shown that L2 learners whose L1 lacks these vowels perceive them as more similar to back vowels than front vowels (*36*). This perceptual assimilation is mirrored in speech production: Productions of /y/ by L2 learners are far less advanced in phonetic space than native speakers’ productions and are indeed often perceived as /u/ by the latter (*37*). The fact that front rounded vowels are readily innovated points to a high ingress rate, while frequent acquisition failure by L2 learners in situations of language contact points to a high egress rate. These facts are inconsistent with the high stability predicted by the phylogenetic method but consistent with our approach, in which high ingress and high egress imply high temperature (Eq. 4).

Similar arguments can be made for the remaining outliers. For instance, all Uralic languages use possessive affixes (e.g., Finnish *auto-ni* “my car,” *auto-si* “your car,” etc.), and the appearance of this system of possession can be dated back to Pre-Proto-Uralic by standard reconstructive techniques (*38*). Possessive affixes are also old in the unrelated Turkic language family (*39*). There is, then, reason to believe that WALS feature 57A on possessive affixes is a false negative of the purely phylogenetic method in (*11*), which classifies possessive affixes as one of the least stable features (Fig. 5). These conclusions are further supported by the fact that an independent method combining a phylogenetic and an areal signal (*9*) agrees with our temperature-based method on three of the four outliers, classifying, like our method but unlike the phylogenetic method, WALS features 11A and 8A as unstable and 57A as stable. There is, in other words, reason to believe that focusing on the phylogenetic signal to the complete exclusion of the areal dimension leads to a number of features being misclassified or mismeasured in terms of their stability and temperature.

## DISCUSSION

Estimating the speed of linguistic change is challenging, essentially, because the signal is poor: Although evolutionary and anthropological evidence suggests that human language in its modern form has existed for at least 100,000 years (*1*), the historical evolution of languages is (necessarily) poorly documented. This documentation only captures a few thousand years for languages with the best coverage, cannot, in principle, go beyond the introduction of the first writing systems, and does not exist at all for the majority of the world’s languages. The rest of the cultural evolution of human language must be reconstructed on the basis of available data; in particular, methods for estimating the temporal stability of features of language have traditionally relied on phylogenetic analysis. Here, we showed that treating language dynamics as a stochastic process combining both a vertical and a horizontal dimension naturally leads to the notion of linguistic temperature, a dimensionless measure of the propensity of linguistic features to undergo change. Temperatures of linguistic features can be readily estimated from purely synchronic information: All that is needed are estimates of feature frequency and isogloss density from a sufficiently large sample of languages and inversion of Eq. 3.

We have offered some evidence in support of our method, in the sense that this method is not liable to some of the false positives and false negatives incurred by some purely phylogenetic methods of stability estimation. Turning now to its limitations, we note that our approach currently only applies to binary features, i.e., features that are either present in or absent from a language. Most genetic methods do not suffer from this limitation: Dediu’s (*11*) procedure, in particular, can be applied to polyvalent and binary features. However, Dediu finds a correlation between estimates for polyvalent and binary (or binarized) features. This suggests that the resolution at which the values of a linguistic variable are recorded may be a minor issue: After all, any polyvalent classification can be reduced to a hierarchy of binary oppositions by a simple translation procedure. Another limitation of our technique is that it treats the evolution of each individual feature independently. Feature interactions are known to exist, however, for example, a language that places objects before verbs is far more likely to also place adverbs before verbs, rather than after them (*40*). It would, in principle, be possible to generalize our method to cater for polyvalent features and feature interactions, by extending the lattice model in the direction of the Axelrod model of cultural dissemination (*41*). The extent to which the behavior of such a generalized model can be characterized analytically is, however, not clear, and temperature estimates may have to be obtained in some other way. Similarly, extending the analysis to multiple summary statistics (beyond feature frequency and isogloss density) is likely to lead to analytical challenges and may necessitate computational inference approaches. Approximate Bayesian computation, for example, is successfully used in population genetics (*42*) and has recently been applied to a comparison of genetic and linguistic evolution (*43*). Other avenues for extending the present model include exploration of transient long-range geographical connections in addition to local spatial effects, incorporation of more realistic selection and mutation dynamics in both the vertical and the horizontal dimension (*44*, *45*), and incorporation of a model of linguistic speciation and a treatment of the resulting geospatial distributions of entire families of languages (*46*).

The derivation of linguistic temperature, together with the empirical demonstration that temperatures of linguistic features are measurable from typological atlases, suggests the existence of large-scale regularities in the transmission of language, in both the vertical and the horizontal dimension. Although the evolutionary trajectories of individual languages are, to a large extent, molded by contingencies of history, when the representation of structural features of language is explored at the level of aggregates of languages, regularities emerge. The estimation of linguistic temperatures is but one possible application resulting from work that combines the mathematical analysis of stochastic systems with modern large-scale linguistic datasets, and we also expect similar approaches to be possible in other areas of cultural evolution outside the narrow domain of language.

## MATERIALS AND METHODS

### Model

The model assumes languages to be distributed among the sites or cells of a regular square lattice and is characterized by five parameters per feature, each a probability: ingress and egress rates in the vertical process (*p*_I_ and *p*_E_), ingress and egress rates in the horizontal process ($p_{I}^{\prime }$ and $p_{E}^{\prime }$), and the relative rate of horizontal versus vertical events (*q*). The model is iterated as follows until statistical equilibrium is reached:

1) Initialize the lattice in a random state (for each feature *F* and lattice cell *C*, *F* is present in *C* with a probability of 0.5).

2) Pick a random cell (language) *C* and a random feature *F*.

3) Execute one of the following steps: (i) With probability 1 − *q*, conduct a vertical event. If *F* is absent from *C*, then acquire *F* with probability *p*_I_ (ingress); if *F* is present in *C*, then lose *F* with probability *p*_E_ (egress). (ii) With probability *q*, conduct a horizontal event. Pick a random nearest-neighbor *C*′ of *C*. If *F* is absent from *C*′, then copy the absence of *F* to *C* with probability $1-p_{I}^{\prime }$; otherwise, set the state of *F* in *C* to “present.” If *F* is present in *C*′, then copy its presence to *C* with probability $1-p_{E}^{\prime }$; otherwise, set the state of *F* in *C* to “absent.”

4) Go to 2.

The stationary distribution of this model may be studied analytically in the special case $p_{I}^{\prime }=p_{E}^{\prime }$ and using numerical simulations in the general case, as detailed in the accompanying Supplementary Materials. More realistic spatial substrates can also be considered in numerical simulations, again as outlined in the Supplementary Materials.

### Empirical estimation of temperatures

To estimate empirical temperatures, the latest (2014) version of the WALS Online database (*32*) was downloaded and used as the empirical basis for measures of feature frequency ρ and isogloss density σ. WALS is a large-scale atlas of structural features of human language, i.e., properties that can be compared across both related and unrelated languages: Examples include aspects of word order, the presence or absence of grammatical items such as articles and inflections, and the presence or absence of different classes of speech sounds. It contains data on varying numbers of features for a total of 2676 languages (approximately a third of the world’s languages). The spatial representation of each language is a latitude-longitude pair, placed approximately in the region of highest speaker density for that language. Language locations in WALS are precolonial and thus represent geospatial distributions before more recent population expansions (*47*).

Since WALS uses a polyvalent coding for most features, a manual pass through the data was first made, retaining only those features that are binary or binarizable on uncontroversial linguistic grounds. Features with fewer than 300 languages in their language sample were discarded to ensure statistically robust results. Sign languages were excluded. This procedure resulted in 35 binary features (see Table 1 for a listing and the Supplementary Materials for detailed information about our binarization scheme).

Temperatures τ were estimated by taking 1000 bootstrap samples for each feature from the feature’s WALS language sample. Feature frequency ρ is the proportion of languages attesting the feature in that feature’s bootstrap sample. In the calculation of isogloss densities σ, the 10 geographically nearest neighbors of each language were considered, found using the haversine formula assuming a perfectly spherical earth. For each language-language pair, an isogloss was recorded if the two languages differed in their value for the feature in question; isogloss density is the number of isoglosses divided by the total number of pairs. Thus, each of the 10 languages in a given language’s neighborhood contributed equally to isogloss density. Variation in the number of nearest neighbors considered did not have an effect on the results (see the Supplementary Materials). Temperatures were recovered by inverting Eq. 3 using a computationally generated hash table, the complete elliptic integral solved numerically using the arithmetic-geometric mean.

To ensure that our method is catching a universal signal about temperatures of linguistic features, rather than contingent properties of particular geographical areas, we performed the two hemispheres test described in (*9*). In this test, the analysis is carried out for the Western and Eastern hemispheres separately, and the temperature estimates arising from the two analyses correlated. As detailed in the Supplementary Materials, the Spearman correlation for temperature estimates in the two hemispheres was found to be 0.52, statistically significant at *P* < 0.01. This compares with the Spearman correlation reported earlier for a genealogical-areal method of stability estimation in the same test, 0.51 (*9*).

### Comparison with phylogenetic method

For the comparison with the phylogenetic method, table S1 to (*11*) was consulted, and only those features were selected for comparison for which our binarization schemes agreed; the PC1 values for the intersecting features were then gathered from table S4 of that publication.

## Supplementary Material

http://advances.sciencemag.org/cgi/content/full/7/1/eabe6540/DC1

Adobe PDF - abe6540_SM.pdf

Geospatial distributions reflect temperatures of linguistic features

## SUPPLEMENTARY MATERIALS

Supplementary material for this article is available at http://advances.sciencemag.org/cgi/content/full/7/1/eabe6540/DC1
