# Supplementary material for: Geospatial distributions reflect temperatures of linguistic features
Source: Sci Adv. 2021 Jan 1;7(1):eabe6540. doi: 10.1126/sciadv.abe6540 (PMC7775759; doi:10.1126/sciadv.abe6540)
Supplement: http://advances.sciencemag.org/cgi/content/full/7/1/eabe6540/DC1 [file supp_7_1_eabe6540__index.html]

Science Advances | Science AdvancesAAASSearchScience AdvancesMenu

## Supplementary Materials

# Geospatial distributions reflect temperatures of linguistic features

Henri Kauhanen, Deepthi Gopal, Tobias Galla, Ricardo Bermúdez-Otero

Download Supplement

**This PDF file includes:**

- Sections S1 to S6
- Figs. S1 to S3
- References

**Files in this Data Supplement:**

- Adobe PDF - abe6540\_SM.pdf
