# Supplementary material for: Geospatial distributions reflect temperatures of linguistic features
Source: Sci Adv. 2021 Jan 1;7(1):eabe6540. doi: 10.1126/sciadv.abe6540 (PMC7775759; doi:10.1126/sciadv.abe6540)
Supplement: http://advances.sciencemag.org/cgi/content/full/7/1/eabe6540/DC1 [file supp_7_1_eabe6540__1.pdf]

Science Advances
AAAS

advances.sciencemag.org/cgi/content/full/7/1/eabe6540/DC1

Supplementary Materials for

Geospatial distributions reflect temperatures of linguistic features

Henri Kauhanen*, Deepthi Gopal, Tobias Galla, Ricardo Bermúdez-Otero

*Corresponding author. Email: henri.kauhanen@uni-konstanz.de

Published 1 January 2021, *Sci. Adv.* **7**, eabe6540 (2021)
DOI: 10.1126/sciadv.abe6540

This PDF file includes:

Sections S1 to S6
Figs. S1 to S3
References

S1 Introduction

This document provides further technical details of the model, its analytical solution, numerical simulations, and the resultant procedure for estimating temperatures of linguistic features from empirical data. We begin by addressing a limited special case of the model, the case where $p'_I = p'_E = 0$, i.e. when the horizontal process is completely faithful. The stationary distribution of this model can be analytically calculated for an isotropic lattice. Details of the calculation are given in Section S2. We then generalize the model first by assuming $p'_I \neq 0$ and $p'_E \neq 0$ but retaining the requirement that $p'_I = p'_E$. This generalization also turns out to yield to an analytical treatment, as detailed in Section S3. In Section S4 we drop the requirement that $p'_I = p'_E$. The full analytical solubility is lost for this more general model, but we show, using numerical simulations, that equations (1)–(4) of the main paper, which supply feature frequency, isogloss density and temperature at the stationary distribution, are valid for this general formulation also. Section S5 reports the results of a number of sanity checks in order to show that our method for evaluating empirical temperatures is robust to (a) choice of geographical area, (b) choice of spatial scale, (c) the spatial structure of the underlying substrate, and (d) potential idiosyncracies arising from the empirical spatial distributions of language families. Finally, Section S6 supplies detailed metadata about the features consulted in our study.

S2 Model: faithful horizontal transmission

To facilitate the analytical exploration of the model's behaviour, we first study the special case $p'_I = p'_E = 0$, i.e. we assume that all horizontal events result in the faithful transmission of a feature from one lattice node to another.

For the purposes of the analytical calculation we will treat the model as a spin system on a two-dimensional regular square lattice with $N = L \times L$ sites and periodic boundary conditions; comparable approaches are available in the literature on voter models (*26*, *27*). We write $s(\mathbf{x}) \in \{-1, 1\}$ for the spin at lattice site $\mathbf{x} = (x_1, x_2)$; $S(\mathbf{x}) = \langle s(\mathbf{x}) \rangle$ for the average spin of $\mathbf{x}$ (over realizations of the stochastic process); and $m = \sum_{\mathbf{x}} S(\mathbf{x})/N$ for the mean magnetization over the entire lattice. The feature frequency ρ, or fraction of up-spins in the system, is related to m by the identity $\rho = (m + 1)/2$. We further write $S(\mathbf{x}, \mathbf{y}) = \langle s(\mathbf{x}) s(\mathbf{y}) \rangle$ for the pair correlation of $s(\mathbf{x})$ and $s(\mathbf{y})$. In summations, $\mathbf{x}'$ is understood to index the set of von Neumann neighbours of site $\mathbf{x}$, i.e. the set

$$\{(x_1 - 1, x_2), (x_1 + 1, x_2), (x_1, x_2 - 1), (x_1, x_2 + 1)\}. \tag{S1}$$

In Sections S2.1–S2.4 below we calculate analytically the stationary-state feature frequency and isogloss density. While we are not aware of existing work in the exact same model, the calculation follows well established principles from the analytical treatments of voter models in statistical mechanics (see (*21*, *27*) and references therein). In particular a very similar calculation is carried out in (*26*). The novelty of our work is therefore not in the calculation itself, but in the application of the analytical calculation to define temperature for linguistic features.

S2.1 Spin flip probability

Central to our analytical derivations is the spin flip probability, i.e. the probability with which the spin at site $\mathbf{x}$ changes its state from -1 to $+1$ or vice versa, if it is selected for potential update. In our model this is of the form

$$w(\mathbf{x}) = (1 - q)A(\mathbf{x}) + qB(\mathbf{x}), \tag{S2}$$

where $A(\mathbf{x})$ is the contribution of the ingress–egress process and $B(\mathbf{x})$ the contribution of the spatial (voter) process. These are

$$A(\mathbf{x}) = \frac{1 - s(\mathbf{x})}{2} p_I + \frac{1 + s(\mathbf{x})}{2} p_E = \frac{1}{2} \left[p_I + p_E + (p_E - p_I) s(\mathbf{x}) \right] \tag{S3}$$

and

$$B(\mathbf{x}) = \frac{1}{4} \sum_{\mathbf{x}'} \frac{1 - s(\mathbf{x}) s(\mathbf{x}')}{2} = \frac{1}{2} \left[1 - \frac{1}{4} \sum_{\mathbf{x}'} s(\mathbf{x}) s(\mathbf{x}') \right], \tag{S4}$$

where it is important to remember that the summation over $\mathbf{x}'$ runs over the four nearest neighbours of $\mathbf{x}$. Hence we have

$$w(\mathbf{x}) = \frac{1 - q}{2} \left[p_I + p_E + (p_E - p_I) s(\mathbf{x}) \right] + \frac{q}{2} \left[1 - \frac{1}{4} \sum_{\mathbf{x}'} s(\mathbf{x}) s(\mathbf{x}') \right]. \tag{S5}$$

S2.2 Stationary-state feature frequency ρ

The spin at $\mathbf{x}$ changes by the amount $-2s(\mathbf{x})$ with probability $\frac{1}{N} w(\mathbf{x})$, the prefactor $1/N$ representing the probability of $\mathbf{x}$ being picked for update. Consequently, the mean spin $S(\mathbf{x}) = \langle s(\mathbf{x}) \rangle$ evolves as

$$S(\mathbf{x}, t + \Delta t) - S(\mathbf{x}, t) = \frac{1}{N} \langle -2w(\mathbf{x}) s(\mathbf{x}) \rangle, \tag{S6}$$

where Δt is the time step associated with each attempted spin flip. Bearing in mind that $s(\mathbf{x}) s(\mathbf{x}) = 1$ and plugging Eq. (S5) in, this implies

$$\frac{S(\mathbf{x}, t + \Delta t) - S(\mathbf{x}, t)}{1/N} = (1 - q) \left[p_I - p_E - (p_I + p_E) S(\mathbf{x}) \right] + q \left[-S(\mathbf{x}) + \frac{1}{4} \sum_{\mathbf{x}'} S(\mathbf{x}') \right]. \tag{S7}$$

Taking the sum over all sites $\mathbf{x}$, one has

$$\begin{aligned} \frac{m(t + \Delta t) - m(t)}{1/N} &= (1 - q)(p_I - p_E) - (1 - q)(p_I + p_E) \frac{1}{N} \sum_{\mathbf{x}} S(\mathbf{x}) - \\ &- \frac{q}{N} \sum_{\mathbf{x}} S(\mathbf{x}) + \frac{q}{4N} \sum_{\mathbf{x}} \sum_{\mathbf{x}'} S(\mathbf{x}'). \end{aligned} \tag{S8}$$

Now $\sum_{\mathbf{x}} \sum_{\mathbf{x}'} S(\mathbf{x}') = 4 \sum_{\mathbf{x}} S(\mathbf{x})$, since the LHS is the sum of the four von Neumann neighbours of all lattice sites, so that each site, having four neighbours, gets counted four times. Using $m = \sum_{\mathbf{x}} S(\mathbf{x})/N$, we then have

$$\frac{m(t + \Delta t) - m(t)}{1/N} = (1 - q) \left[p_I - p_E - (p_I + p_E) m \right]. \tag{S9}$$

With the standard choice $\Delta t = 1/N$, and taking the limit $N \to \infty$ (i.e. the continuous-time limit $\Delta t \to 0$), we thus find

$$\frac{dm}{dt} = (1 - q) \left[p_I - p_E - (p_I + p_E) m \right]. \tag{S10}$$

Hence the mean magnetization in the stationary state ($dm/dt = 0$) is

$$m = \frac{p_I - p_E}{p_I + p_E}. \tag{S11}$$

From this, using $\rho = (m + 1)/2$, we find

$$\rho = \frac{p_I}{p_I + p_E} \tag{S12}$$

for the fraction of up-spins in the stationary state.

S2.3 Pair correlation function

To compute the pair correlation $S(\mathbf{x}, \mathbf{y}) = \langle s(\mathbf{x})s(\mathbf{y})\rangle$, we note that $s(\mathbf{x})s(\mathbf{y})$ changes by the amount $-2s(\mathbf{x})s(\mathbf{y})$ if either $\mathbf{x}$ or $\mathbf{y}$ flips spin. Assuming $\mathbf{x} \neq \mathbf{y}$, and working directly in the continuous-time limit, we have

$$\frac{dS(\mathbf{x}, \mathbf{y})}{dt} = \langle -2\left[w(\mathbf{x}) + w(\mathbf{y})\right] s(\mathbf{x})s(\mathbf{y})\rangle. \tag{S13}$$

After some algebra we find

$$\begin{aligned}\frac{dS(\mathbf{x}, \mathbf{y})}{dt} &= (1-q)\left[(p_I - p_E)[S(\mathbf{x}) + S(\mathbf{y})] - 2(p_I + p_E)S(\mathbf{x}, \mathbf{y})\right] + \\ &+ q\left[-2S(\mathbf{x}, \mathbf{y}) + \frac{1}{4}\sum_{\mathbf{x}'} S(\mathbf{x}', \mathbf{y}) + \frac{1}{4}\sum_{\mathbf{y}'} S(\mathbf{x}, \mathbf{y}')\right],\end{aligned} \tag{S14}$$

where the summation over $\mathbf{y}'$ is over the four nearest neighbours of $\mathbf{y}$.

We now assume translation invariance and write $C(\mathbf{r}) = C(\mathbf{x}-\mathbf{y}) = S(\mathbf{x}, \mathbf{y})$. Then, for $\mathbf{r} \neq \mathbf{0}$,

$$\begin{aligned}\frac{dC(\mathbf{r})}{dt} &= (1-q)\left[(p_I - p_E)[S(\mathbf{x}) + S(\mathbf{y})] - 2(p_I + p_E)C(\mathbf{r})\right] + \\ &+ q\left[-2C(\mathbf{r}) + \frac{1}{4}\sum_{\mathbf{x}'} C(\mathbf{x}' - \mathbf{y}) + \frac{1}{4}\sum_{\mathbf{y}'} C(\mathbf{x} - \mathbf{y}')\right].\end{aligned} \tag{S15}$$

Due to translation invariance the two summations on the RHS coincide, and we have (always restricting $\mathbf{r} \neq \mathbf{0}$)

$$\frac{dC(\mathbf{r})}{dt} = (1-q)\left[(p_I - p_E)[S(\mathbf{x}) + S(\mathbf{y})] - 2(p_I + p_E)C(\mathbf{r})\right] + 2q\Delta C(\mathbf{r}), \tag{S16}$$

where Δ is the lattice Laplace operator

$$\Delta f(\mathbf{x}) = -f(\mathbf{x}) + \frac{1}{4}\sum_{\mathbf{x}'} f(\mathbf{x}'). \tag{S17}$$

We note that we always have the boundary condition $C(\mathbf{0}, t) = 1$ for all t (self-correlation is 1 at all times, as $s(\mathbf{x})^2 = 1$).

S2.4 Stationary-state isogloss density σ

If $C(\mathbf{e}_1, t)$ were known, where $\mathbf{e}_1$ is the unit vector $(1, 0)$, the isogloss density could be obtained via the identity

$$\sigma(t) = \frac{1 - C(\mathbf{e}_1, t)}{2}. \tag{S18}$$

Thus, knowing the limiting ($t \to \infty$) value of $C(\mathbf{e}_1)$ would imply the stationary-state isogloss density σ.

At the stationary state, $S(\mathbf{x}) + S(\mathbf{y}) = 2m$ and $\frac{dC(\mathbf{r})}{dt} = 0$. Assuming $\mathbf{r} \neq \mathbf{0}$, Eq. (S16) then implies

$$0 = (1-q)\left[2(p_I - p_E)m - 2(p_I + p_E)C(\mathbf{r})\right] + 2q\Delta C(\mathbf{r}), \tag{S19}$$

in other words,

$$\begin{aligned} 0 &= (1-q)\left[(p_I - p_E)m - (p_I + p_E)C(\mathbf{r})\right] + q\Delta C(\mathbf{r}) \\ &= (1-q)(p_I+p_E)\left[\frac{p_I - p_E}{p_I + p_E}m - C(\mathbf{r})\right] + q\Delta C(\mathbf{r}) \\ &= (1-q)(p_I+p_E)\left[m^2 - C(\mathbf{r})\right] + q\Delta C(\mathbf{r}). \end{aligned} \tag{S20}$$

In the special case $q = 0$ (i.e., no spatial interaction between neighbouring sites), this implies $C(\mathbf{r}) = m^2$ for all $\mathbf{r} \neq \mathbf{0}$. Thus for any two sites $\mathbf{x} \neq \mathbf{y}$, $\langle s(\mathbf{x})s(\mathbf{y})\rangle = m^2$, indicating that spins at different sites are fully uncorrelated. This is what one would expect, as all sites operate independently when $q = 0$.

In the special case $q = 1$ (no ingress–egress dynamics within the sites), on the other hand, we obtain $\Delta C(\mathbf{r}) = 0$ for $\mathbf{r} \neq 0$. We also have $C(\mathbf{0}) = 0$. This implies $C(\mathbf{r}) = 1$ everywhere, so either all spins are up or all are down. This is the only possible stationary state when the only dynamics is through nearest-neighbour interactions.

Now suppose $0 < q < 1$. Dividing Eq. (S20) by q we obtain

$$\begin{aligned} 0 &= \frac{1-q}{q}(p_I+p_E)\left[m^2 - C(\mathbf{r})\right] + \Delta C(\mathbf{r}) \\ &= \frac{(q-1)(p_I+p_E)}{q}\left[C(\mathbf{r}) - m^2\right] + \Delta C(\mathbf{r}) \\ &= \alpha C(\mathbf{r}) - \alpha m^2 + \Delta C(\mathbf{r}), \end{aligned} \tag{S21}$$

where we write

$$\alpha = \frac{(q-1)(p_I+p_E)}{q}. \tag{S22}$$

Now let

$$c(\mathbf{r}) = C(\mathbf{r}) - m^2. \tag{S23}$$

We note that $\Delta C(\mathbf{r}) = \Delta c(\mathbf{r})$. To solve Eq. (S21), it then suffices to solve (for $\mathbf{r} \neq \mathbf{0}$)

$$\Delta c(\mathbf{r}) + \alpha c(\mathbf{r}) = 0 \tag{S24}$$

subject to the condition

$$c(\mathbf{0}) = 1 - m^2. \tag{S25}$$

We now first focus on the equation

$$\Delta G(\mathbf{r}) + \alpha G(\mathbf{r}) = -\delta_{\mathbf{r},\mathbf{0}}, \tag{S26}$$

for all $\mathbf{r}$ (including $\mathbf{r} = \mathbf{0}$), and where $\delta_{\mathbf{x},\mathbf{y}}$ is the Kronecker delta.

Let $G_\alpha(\mathbf{r})$ be a solution of Eq. (S26). Then

$$c(\mathbf{r}) = (1-m^2)\frac{G_\alpha(\mathbf{r})}{G_\alpha(\mathbf{0})} \tag{S27}$$

is a solution of Eqs. (S24) and (S25). This can be seen as follows: first, from Eq. (S27),

$$c(\mathbf{0}) = (1-m^2)\frac{G_\alpha(\mathbf{0})}{G_\alpha(\mathbf{0})} = 1 - m^2, \tag{S28}$$

so the condition in Eq. (S25) is met. Second, we need to show that Eqs. (S26) and (S27) imply $\Delta c(\mathbf{r}) + \alpha c(\mathbf{r}) = 0$ for $\mathbf{r} \neq \mathbf{0}$. For $\mathbf{r} \neq \mathbf{0}$ we have

$$\Delta G(\mathbf{r}) + \alpha G(\mathbf{r}) = 0 \tag{S29}$$

from Eq. (S26). The quantity $c(\mathbf{r})$ in Eq. (S27) is proportional to $G_\alpha(\mathbf{r})$ with a proportionality constant $(1 - m^2)/G_\alpha(\mathbf{0})$ which is independent of $\mathbf{r}$. Given that $G_\alpha(\mathbf{r})$ fulfills Eq. (S29) for $\mathbf{r} \neq \mathbf{0}$ it is then clear that $c(\mathbf{r})$ fulfills $\Delta c(\mathbf{r}) + \alpha c(\mathbf{r}) = 0$ for $\mathbf{r} \neq \mathbf{0}$, i.e. Eq. (S24).

So we are left with the task of finding a solution of

$$\Delta G(\mathbf{r}) + \alpha G(\mathbf{r}) = -\delta_{\mathbf{r},\mathbf{0}}. \tag{S30}$$

Let us write $G(\mathbf{r})$ in Fourier representation:

$$G(\mathbf{r}) = \frac{1}{(2\pi)^2} \int_{-\pi}^{\pi} \int_{-\pi}^{\pi} dk_1 dk_2 \; e^{i\mathbf{k}\cdot\mathbf{r}} \widehat{G}(\mathbf{k}), \tag{S31}$$

where $\mathbf{k} = (k_1, k_2)$ and $\widehat{G}(\mathbf{k})$ is the Fourier transform of $G(\mathbf{r})$, i.e.

$$\widehat{G}(\mathbf{k}) = \sum_{\mathbf{r}} e^{-i\mathbf{k}\cdot\mathbf{r}} G(\mathbf{r}) = \sum_x \sum_y e^{-ixk_1 - iyk_2} G(x, y). \tag{S32}$$

Applying this to both sides of Eq. (S30) leads to

$$\sum_{x,y} e^{-ixk_1 - iyk_2} \left[\Delta G(\mathbf{r}) + \alpha G(\mathbf{r})\right] = -1. \tag{S33}$$

Next, notice

$$\begin{aligned}
\sum_{x,y} e^{-ixk_1 - iyk_2} \Delta G(x, y) &= \frac{1}{4} \sum_{x,y} e^{-ixk_1 - iyk_2} \left[G(x + 1, y) + G(x - 1, y) + \right. \\
&\qquad \left. + G(x, y + 1) + G(x, y - 1) - 4G(x, y)\right] \\
&= \frac{1}{4} \sum_{x,y} \left[e^{-i(x-1)k_1 - iyk_2} + e^{-i(x+1)k_1 - iyk_2} + \right. \\
&\qquad \left. + e^{-ixk_1 - i(y-1)k_2} + e^{-ixk_1 - i(y+1)k_2} - 4e^{-ixk_1 - iyk_2}\right] G(x, y) \\
&= \frac{1}{4} \sum_{x,y} \left[\underbrace{e^{ik_1} + e^{-ik_1}}_{=2\cos k_1} + \underbrace{e^{ik_2} + e^{-ik_2}}_{=2\cos k_2} - 4\right] e^{-ixk_1 - iyk_2} G(x, y) \\
&= \frac{1}{2} \left[\cos k_1 + \cos k_2 - 2\right] \sum_{x,y} e^{-ixk_1 - iyk_2} G(x, y) \\
&= \frac{1}{2} \left[\cos k_1 + \cos k_2 - 2\right] \widehat{G}(\mathbf{k}).
\end{aligned} \tag{S34}$$

Using this in Eq. (S33) gives

$$\left[\frac{1}{2} \left(\cos k_1 + \cos k_2 - 2\right) + \alpha\right] \widehat{G}(\mathbf{k}) = -1, \tag{S35}$$

in other words

$$\widehat{G}(\mathbf{k}) = \frac{1}{1 - \alpha - \frac{1}{2}\left[\cos k_1 + \cos k_2\right]}. \tag{S36}$$

Using Eq. (S31) we then have

$$G_\alpha(\mathbf{r}) = \frac{1}{(2\pi)^2} \int_{-\pi}^{\pi} \int_{-\pi}^{\pi} \frac{e^{i\mathbf{k}\cdot\mathbf{r}} dk_1 dk_2}{1 - \alpha - \frac{1}{2}(\cos k_1 + \cos k_2)}. \tag{S37}$$

Hence

$$G_\alpha(\mathbf{0}) = \frac{1}{(2\pi)^2} \int_{-\pi}^{\pi} \int_{-\pi}^{\pi} \frac{dk_1 dk_2}{1 - \alpha - \frac{1}{2}(\cos k_1 + \cos k_2)}. \tag{S38}$$

The integrand is symmetric with respect to $k_1 \leftrightarrow -k_1$ and $k_2 \leftrightarrow -k_2$ respectively, due to the identity $\cos(k) = \cos(-k)$. Hence

$$\begin{aligned} G_\alpha(\mathbf{0}) &= \frac{1}{\pi^2} \int_{0}^{\pi} \int_{0}^{\pi} \frac{dk_1 dk_2}{1 - \alpha - \frac{1}{2}(\cos k_1 + \cos k_2)} \\ &= \frac{1}{1-\alpha} \frac{1}{\pi^2} \int_{0}^{\pi} \int_{0}^{\pi} \frac{dk_1 dk_2}{1 - \frac{1}{2(1-\alpha)}(\cos k_1 + \cos k_2)}. \end{aligned} \tag{S39}$$

This expression is related to the complete elliptic integral of the first kind (*48*). We use the following notation:

$$K(z) = \frac{1}{2\pi} \int_{0}^{\pi} du \int_{0}^{\pi} dv \frac{1}{1 - \frac{z}{2}(\cos u + \cos v)}. \tag{S40}$$

Hence we have

$$G_\alpha(\mathbf{0}) = \frac{2}{\pi(1-\alpha)} K\left(\frac{1}{1-\alpha}\right) = \frac{2K_\alpha}{\pi(1-\alpha)}, \tag{S41}$$

where we abbreviate

$$K_\alpha = K\left(\frac{1}{1-\alpha}\right) \tag{S42}$$

for convenience.

Next, we write the Laplacian $\Delta G_\alpha(\mathbf{0})$ in full:

$$\Delta G_\alpha(\mathbf{0}) = -G_\alpha(\mathbf{0}) + \frac{1}{4}[G_\alpha(1,0) + G_\alpha(-1,0) + G_\alpha(0,1) + G_\alpha(0,-1)]. \tag{S43}$$

Assuming isotropy, each term in the square brackets is equal to $G_\alpha(\mathbf{e}_1) = G_\alpha(1,0)$. Hence Eq. (S30), evaluated at $\mathbf{r} = \mathbf{0}$, takes the form

$$G_\alpha(\mathbf{e}_1) + (\alpha - 1)G_\alpha(\mathbf{0}) = -1, \tag{S44}$$

in other words

$$G_\alpha(\mathbf{0}) - G_\alpha(\mathbf{e}_1) = 1 + \alpha G_\alpha(\mathbf{0}). \tag{S45}$$

From this we find

$$1 - \frac{G_\alpha(\mathbf{e}_1)}{G_\alpha(\mathbf{0})} = \left[\alpha + \frac{1}{G_\alpha(\mathbf{0})}\right]. \tag{S46}$$

Now using Eqs. (S23) and (S27),

$$C(\mathbf{r}) = m^2 + c(\mathbf{r}) = m^2 + (1 - m^2)\frac{G_\alpha(\mathbf{r})}{G_\alpha(\mathbf{0})}. \tag{S47}$$

The stationary-state isogloss density σ is then

$$\begin{aligned}\sigma &= \frac{1}{2}[1 - C(\mathbf{e}_1)] \\ &= \frac{1}{2}\left[1 - m^2 - (1 - m^2)\frac{G_\alpha(\mathbf{e}_1)}{G_\alpha(\mathbf{0})}\right] \\ &= \frac{1}{2}(1 - m^2)\left[1 - \frac{G_\alpha(\mathbf{e}_1)}{G_\alpha(\mathbf{0})}\right] \\ &= \frac{1}{2}(1 - m^2)\left[\alpha + \frac{1}{G_\alpha(\mathbf{0})}\right] \\ &= \frac{1}{2}(1 - m^2)\left[\alpha + \frac{\pi(1-\alpha)}{2K_\alpha}\right],\end{aligned} \tag{S48}$$

where we have used Eq. (S41). On the other hand,

$$1 - m^2 = 1 - \frac{(p_I - p_E)^2}{(p_I + p_E)^2} = 4\left(\frac{p_I}{p_I + p_E}\right)\left(\frac{p_E}{p_I + p_E}\right) = 4\rho(1-\rho), \tag{S49}$$

so that

$$\sigma = 2\rho(1-\rho)\left[\alpha + \frac{\pi(1-\alpha)}{2K_\alpha}\right]. \tag{S50}$$

Recalling that

$$\alpha = -\tau = -\frac{(1-q)(p_I + p_E)}{q}, \tag{S51}$$

see Eq. (S22), and defining $\tau = -\alpha$, i.e. $K_\alpha = K_{-\tau}$, yields our final equation for the stationary-state isogloss density:

$$\sigma = 2H(\tau)\rho(1-\rho), \tag{S52}$$

where

$$H(\tau) = \frac{\pi(1+\tau)}{2K\left(\frac{1}{1+\tau}\right)} - \tau. \tag{S53}$$

The expression in Eq. (S52) is a downward-opening parabola in ρ whose height is fixed by $H(\tau)$, where

$$\tau = \frac{(1-q)(p_I + p_E)}{q}. \tag{S54}$$

S2.5 Sanity check: the limits $\tau \to 0$ and $\tau \to \infty$

The complete elliptic integral $K(z)$ has the following known properties (*49*):

$$\lim_{z\to 0} K(z) = \frac{\pi}{2} \quad \text{and} \quad \lim_{z\to 1} K(z) = \infty. \tag{S55}$$

Now consider the limit $\tau \to 0$. This limit is relevant when either $q \to 1$, or both $p_E \to 0$ and $p_I \to 0$, see Eq. (S54). These are situations in which the spatial (voter) process dominates the ingress–egress dynamics. In this limit $1/(1+\tau) \to 1$, so that $K_{-\tau} = K(1/(1+\tau)) \to \infty$. Noting that Eq. (S53) can be written in the form

$$H(\tau) = \tau\left[\frac{\pi}{2K_{-\tau}} - 1\right] + \frac{\pi}{2K_{-\tau}}, \tag{S56}$$

we then find that $H(\tau) \to 0$. Thus, in the limit where the spatial (voter) process completely overtakes the ingress–egress process, the stationary-state isogloss density is zero, indicating that all sites agree in their spin.

Next consider $\tau \to \infty$. This limit occurs when $p_I + p_E > 0$ and $q \to 0$, i.e. the ingress–egress process dominates. Then $1/(1+\tau) \to 0$, so that $K_{-\tau} \to \pi/2$. From Eq. (S56), we then find that $H(\tau) \to 1$ in this case. Thus, in the limit where the ingress–egress process completely overtakes the spatial process, the stationary-state isogloss density is given by the parabola $\sigma = 2\rho(1-\rho)$, indicating complete independence of the individual spins.

S3 Model: undirected horizontal error

We now drop the requirement that p'_I and p'_E be zero, but retain the assumption that $p'_I = p'_E$, i.e. that errors in the horizontal process are equally likely to innovate a feature as they are to lose it.

S3.1 Spin flip probability

The spin flip rate associated with the (vertical) dynamics within a site is not affected by the generalization, and remains as before

$$A(\mathbf{x}) = \frac{1-s(\mathbf{x})}{2} p_I + \frac{1+s(\mathbf{x})}{2} p_E = \frac{1}{2}\left[p_I + p_E + (p_E - p_I)s(\mathbf{x})\right]. \tag{S57}$$

The rate with which the spin at site $\mathbf{x}$ flips due to interactions with one of the neighbours changes though due to the modification of the model. We now have

$$\begin{aligned} B(\mathbf{x}) = \frac{1}{4}\sum_{\mathbf{x}'}\Bigg[&\frac{1+s(\mathbf{x})}{2}\frac{1+s(\mathbf{x}')}{2}p'_I + \frac{1+s(\mathbf{x})}{2}\frac{1-s(\mathbf{x}')}{2}(1-p'_I) + \\ &+\frac{1-s(\mathbf{x})}{2}\frac{1+s(\mathbf{x}')}{2}(1-p'_E) + \frac{1-s(\mathbf{x})}{2}\frac{1-s(\mathbf{x}')}{2}p'_E\Bigg]. \end{aligned} \tag{S58}$$

The first term only contributes if $s(\mathbf{x}) = s(\mathbf{x}') = -1$. Without copying errors, no change of $s(\mathbf{x})$ would occur. However, with probability p'_I there is an error the attempted copying of $s(\mathbf{x}') = -1$, and $s(\mathbf{x})$ flips from $s(\mathbf{x}) = -1$ to $s(\mathbf{x}) = 1$. The second term contributes only when $s(\mathbf{x}) = 1$ and $s(\mathbf{x}') = -1$. This only results in a flip of $s(\mathbf{x})$ (from 1 to -1) if no error is made in the copying process, i.e. if there is no ingress event. Hence the prefactor $1 - p'_I$. With probability p'_I the copying is associated with an ingress-type error, and $s(\mathbf{x})$ remains at $s = -1$, and hence no flip occurs. The third and fourth terms have a similar interpretation.

We now re-organise terms in Eq. (S58):

$$\begin{aligned} B(\mathbf{x}) &= \frac{1}{4}\sum_{\mathbf{x}'}\Bigg[\frac{1+s(\mathbf{x})}{2}\frac{1-s(\mathbf{x}')}{2} + \frac{1-s(\mathbf{x})}{2}\frac{1+s(\mathbf{x}')}{2} + \\ &\quad +p'_I\left(\frac{1+s(\mathbf{x})}{2}\frac{1+s(\mathbf{x}')}{2} - \frac{1+s(\mathbf{x})}{2}\frac{1-s(\mathbf{x}')}{2}\right) + \\ &\quad +p'_E\left(\frac{1-s(\mathbf{x})}{2}\frac{1-s(\mathbf{x}')}{2} - \frac{1-s(\mathbf{x})}{2}\frac{1+s(\mathbf{x}')}{2}\right)\Bigg] \\ &= \frac{1}{4}\sum_{\mathbf{x}'}\left[\frac{1-s(\mathbf{x})s(\mathbf{x}')}{2} + p'_I\frac{s(\mathbf{x}')+s(\mathbf{x})s(\mathbf{x}')}{2} + p'_E\frac{s(\mathbf{x})s(\mathbf{x}')-s(\mathbf{x}')}{2}\right] \\ &= \frac{1}{4}\sum_{\mathbf{x}'}\left[\frac{1-s(\mathbf{x})s(\mathbf{x}')}{2} + (p'_I+p'_E)\frac{s(\mathbf{x})s(\mathbf{x}')}{2} + (p'_I-p'_E)\frac{s(\mathbf{x}')}{2}\right]. \end{aligned} \tag{S59}$$

We therefore have

$$A(\mathbf{x}) = \frac{1 - s(\mathbf{x})}{2} p_I + \frac{1 + s(\mathbf{x})}{2} p_E = \frac{1}{2} \left[p_I + p_E + (p_E - p_I) s(\mathbf{x}) \right] \tag{S60}$$

as in the original model, and

$$B(\mathbf{x}) = \frac{1}{2} \left[1 - \frac{1}{4} \sum_{\mathbf{x}'} \left\{ s(\mathbf{x}) s(\mathbf{x}') - (p'_I + p'_E) s(\mathbf{x}) s(\mathbf{x}') - (p'_I - p'_E) s(\mathbf{x}') \right\} \right], \tag{S61}$$

where it is important to remember that the summation over $\mathbf{x}'$ runs over the four nearest neighbours of $\mathbf{x}$. The total rate with which the spin at site $\mathbf{x}$ flips is therefore

$$\begin{aligned} w(\mathbf{x}) = & \frac{1-q}{2} \left[p_I + p_E + (p_E - p_I) s(\mathbf{x}) \right] + \\ & + \frac{q}{2} \left[1 - \frac{1}{4} \sum_{\mathbf{x}'} \left\{ s(\mathbf{x}) s(\mathbf{x}') - (p'_I + p'_E) s(\mathbf{x}) s(\mathbf{x}') - (p'_I - p'_E) s(\mathbf{x}') \right\} \right]. \end{aligned} \tag{S62}$$

S3.2 Stationary-state feature frequency ρ

As in the more basic model, we proceed to compute the magnetization in the stationary state, or equivalently the number of spins in state +1 (the fraction of sites where the feature is present). Similar to Eq. (S6) we have

$$\begin{aligned} \frac{S(\mathbf{x}, t + \Delta t) - S(\mathbf{x}, t)}{1/N} & = \langle -2 w(\mathbf{x}) s(\mathbf{x}) \rangle \\ & = (1-q) \left[p_I - p_E - (p_I + p_E) S(\mathbf{x}) \right] + q \Bigg[- S(\mathbf{x}) + \\ & \quad + \frac{1}{4} \sum_{\mathbf{x}'} \left\{ S(\mathbf{x}') - (p'_I + p'_E) S(\mathbf{x}') - (p'_I - p'_E) \langle s(\mathbf{x}) s(\mathbf{x}') \rangle \right\} \Bigg]. \end{aligned} \tag{S63}$$

It is now difficult to proceed for the case of general p'_I and p'_E. The difficulty arises from the last term in Eq. (S63), which contains a correlation function. This means that the equation for the mean magnetisation is not closed. Similarly, if we proceeded to calculate the correlation function, we would meet averages of objects involving three different sites.

However, we can continue if we set $p'_I = p'_E$. The problematic term then vanishes. Assuming $p'_I = p'_E$ means that errors in the copying process from neighbours occur independent of what spin state is copied. We write $\lambda = p'_I = p'_E$ and always assume $0 \le \lambda < 1/2$. The case $\lambda = 0$ describes error-free horizontal transmission. For $\lambda = 1/2$ all information is lost in the transmission, the received state is entirely random (feature present or absent, each with probability $1/2$), and independent of the transmitted state. The restriction $\lambda < 1/2$ therefore encapsulates the assumption that some information is transmitted on average. A choice of $\lambda > 1/2$ would reflect situations in which there is a tendency for bits to become actively inverted in transmission ('worse than random'). We do not study these here.

Taking the sum over all sites $\mathbf{x}$ in Eq. (S63), one now finds

$$\begin{aligned} \frac{m(t + \Delta t) - m(t)}{1/N} = & (1-q)(p_I - p_E) - (1-q)(p_I + p_E) \frac{1}{N} \sum_{\mathbf{x}} S(\mathbf{x}) - \\ & - \frac{q}{N} \sum_{\mathbf{x}} S(\mathbf{x}) + \frac{q}{4N} (1 - 2\lambda) \sum_{\mathbf{x}} \sum_{\mathbf{x}'} S(\mathbf{x}'). \end{aligned} \tag{S64}$$

From this in turn we obtain

$$
\begin{aligned}
\frac{dm}{dt} &= (1-q)\,[p_I - p_E - (p_I + p_E)m] - 2q\lambda m \\
&= (1-q)(p_I - p_E) + m[-(1-q)(p_I + p_E) - 2q\lambda].
\end{aligned} \tag{S65}
$$

Hence in the stationary state

$$
m = \frac{(p_I - p_E)}{p_I + p_E + 2\frac{q}{1-q}\lambda}. \tag{S66}
$$

Comparing this to Eq. (S11) we note the additional term proportional to λ in the denominator. This term drives m to zero. This makes sense, as errors in the copying process from neighbouring sites 'equalize' the frequencies of the two spin states present in the system. Suppose for example there is a majority of +1 spins. Then an attempted copying event is more likely to be one in which a +1 spin is copied. Errors in this process reduce the fraction of +1 spins, and increase the fraction of −1 spins.

Using $\rho = (m+1)/2$, we find from Eq. (S66):

$$
\rho = \frac{1}{2}\frac{p_I - p_E + p_I + p_E + 2\frac{q}{1-q}\lambda}{p_I + p_E + 2\frac{q}{1-q}\lambda} = \frac{(1-q)p_I + q\lambda}{(1-q)(p_I + p_E) + 2q\lambda}. \tag{S67}
$$

This quantity is manifestly between 0 and 1 (numerator and denominator are both non-negative, and the denominator is always greater than or equal to the numerator). For $\lambda = 0$ this reduces to Eq. (S12), the formula for the simpler model. We also notice that $\rho = 1/2$ when $q = 1$. This makes sense, there is then only the horizontal process, and copying errors happen with the same rate λ, independently of the state that is copied (absence or presence of the feature). As mentioned above, this drives the system to equal fractions of −1 and +1 spins.

S3.3 Correlation function and isogloss density

We start from [see Eq. (S13)]

$$
\frac{dS(\mathbf{x},\mathbf{y})}{dt} = \langle -2\,[w(\mathbf{x}) + w(\mathbf{y})]\,s(\mathbf{x})s(\mathbf{y})\rangle, \tag{S68}
$$

where now

$$
\begin{aligned}
w(\mathbf{x}) &= \frac{1-q}{2}\,[p_I + p_E + (p_E - p_I)s(\mathbf{x})] + \frac{q}{2}\left[1 - \frac{1}{4}\sum_{\mathbf{x}'}\{s(\mathbf{x})s(\mathbf{x}') - 2\lambda s(\mathbf{x})s(\mathbf{x}')\}\right] \\
&= \frac{1-q}{2}\,[p_I + p_E + (p_E - p_I)s(\mathbf{x})] + \frac{q}{2}\left[1 - \frac{1}{4}(1-2\lambda)\sum_{\mathbf{x}'} s(\mathbf{x})s(\mathbf{x}')\right].
\end{aligned} \tag{S69}
$$

From this we find

$$
\begin{aligned}
\frac{dS(\mathbf{x},\mathbf{y})}{dt} &= (1-q)\,[(p_I - p_E)[S(\mathbf{x}) + S(\mathbf{y})] - 2(p_I + p_E)S(\mathbf{x},\mathbf{y})] + \\
&\quad + q\left[-2S(\mathbf{x},\mathbf{y}) + \frac{1}{4}(1-2\lambda)\sum_{\mathbf{x}'} S(\mathbf{x}',\mathbf{y}) + \frac{1}{4}(1-2\lambda)\sum_{\mathbf{y}'} S(\mathbf{x},\mathbf{y}')\right],
\end{aligned} \tag{S70}
$$

where the summation over $\mathbf{y}'$ is over the four nearest neighbours of $\mathbf{y}$.

As in the analysis of the simpler model we now assume translation invariance and write $C(\mathbf{r}) = C(\mathbf{x}-\mathbf{y}) = S(\mathbf{x},\mathbf{y})$. Then, for $\mathbf{r} \neq \mathbf{0}$,

$$\begin{aligned}\frac{dC(\mathbf{r})}{dt} &= (1-q)\left[(p_I - p_E)[S(\mathbf{x}) + S(\mathbf{y})] - 2(p_I + p_E)C(\mathbf{r})\right] + \\ &+ q\left[-2C(\mathbf{r}) + \frac{1}{4}(1-2\lambda)\sum_{\mathbf{x}'} C(\mathbf{x}'-\mathbf{y}) + \frac{1}{4}(1-2\lambda)\sum_{\mathbf{y}'} C(\mathbf{x}-\mathbf{y}')\right].\end{aligned} \tag{S71}$$

Adding and subtracting a term (the aim is to isolate a lattice Laplacian) we obtain

$$\begin{aligned}\frac{dC(\mathbf{r})}{dt} &= (1-q)\left[(p_I - p_E)[S(\mathbf{x}) + S(\mathbf{y})] - 2\left(p_I + p_E + 2\frac{q}{1-q}\lambda\right)C(\mathbf{r})\right] + \\ &+ q\left[-2(1-2\lambda)C(\mathbf{r}) + \frac{1}{4}(1-2\lambda)\sum_{\mathbf{x}'} C(\mathbf{x}'-\mathbf{y}) + \frac{1}{4}(1-2\lambda)\sum_{\mathbf{y}'} C(\mathbf{x}-\mathbf{y}')\right].\end{aligned} \tag{S72}$$

Due to translation invariance the two summations on the RHS coincide, and we have (always restricting $\mathbf{r} \neq \mathbf{0}$)

$$\begin{aligned}\frac{dC(\mathbf{r})}{dt} &= (1-q)\left[(p_I - p_E)[S(\mathbf{x}) + S(\mathbf{y})] - 2\left(p_I + p_E + 2\frac{q}{1-q}\lambda\right)C(\mathbf{r})\right] + \\ &+ 2q(1-2\lambda)\Delta C(\mathbf{r}),\end{aligned} \tag{S73}$$

where Δ is the lattice Laplacian.

In the stationary state, $S(\mathbf{x}) + S(\mathbf{y}) = 2m$ and $\frac{dC(\mathbf{r})}{dt} = 0$. Assuming $\mathbf{r} \neq \mathbf{0}$, we then obtain the following from Eq. (S73):

$$0 = (1-q)\left[2(p_I - p_E)m - 2\left(p_I + p_E + 2\frac{q}{1-q}\lambda\right)C(\mathbf{r})\right] + 2q(1-2\lambda)\Delta C(\mathbf{r}). \tag{S74}$$

In other words,

$$0 = (1-q)\left(p_I + p_E + 2\frac{q}{1-q}\lambda\right)\left[m^2 - C(\mathbf{r})\right] + q(1-2\lambda)\Delta C(\mathbf{r}). \tag{S75}$$

In the special case $q = 0$ (i.e., no spatial interaction between neighbouring sites), this implies $C(\mathbf{r}) = m^2$ for all $\mathbf{r} \neq \mathbf{0}$, as in the simpler model (but note that the stationary value of m is different in the two models).

We note that the term containing the lattice Laplacian does not contribute when either $q = 0$ or $\lambda = 1/2$. If $q = 0$, then there is no copying process from neighbouring sites in the model from the beginning, so there is no interaction at all between the different sites. As mentioned above, if $\lambda = 1/2$ no information is transferred in copying processes from neighbours, as the state to be copied is transferred faithfully with probability or inverted, each with probability $1/2$. This does not constitute any meaningful interaction, and neither does it create correlations between sites. The absence of the Laplacian reflects this.

In the following we will always exclude such pathological cases, and assume $0 < q < 1$ and $\lambda \neq \frac{1}{2}$. Dividing Eq. (S75) by $q(1-2\lambda)$ we obtain

$$\begin{aligned}0 &= \frac{q-1}{q(1-2\lambda)}\left(p_I + p_E + 2\frac{q}{1-q}\lambda\right)\left[C(\mathbf{r}) - m^2\right] + \Delta C(\mathbf{r}) \\ &= \alpha C(\mathbf{r}) - \alpha m^2 + \Delta C(\mathbf{r}),\end{aligned} \tag{S76}$$

with

$$\alpha = \frac{q-1}{q(1-2\lambda)} \left(p_I + p_E + 2\frac{q}{1-q}\lambda \right). \tag{S77}$$

This is of the same form as Eq. (S21), except that the expression for α has changed [cf. Eq. (S22)].

We can therefore use all results obtained for the simpler model, provided we use the expression for α in Eq. (S77). In particular we have

$$\sigma = 2\rho(1-\rho) \left[\alpha + \frac{\pi(1-\alpha)}{2K_\alpha} \right]. \tag{S78}$$

as before [cf. Eq. (S50)], but where α is given in Eq. (S77). Defining again $\tau = -\alpha$, i.e. $K_\alpha = K_{-\tau}$, we have again [cf. Eq. (S52)]:

$$\sigma = 2H(\tau)\rho(1-\rho), \tag{S79}$$

where

$$H(\tau) = \frac{\pi(1+\tau)}{2K\left(\frac{1}{1+\tau}\right)} - \tau. \tag{S80}$$

The height of the downward-opening parabola in ρ in Eq. (S79) is fixed by $H(\tau)$, where

$$\tau = -\alpha = \frac{1-q}{q(1-2\lambda)} \left(p_I + p_E + 2\frac{q}{1-q}\lambda \right), \tag{S81}$$

i.e.,

$$\tau = \frac{(1-q)(p_I + p_E) + 2q\lambda}{q(1-2\lambda)}. \tag{S82}$$

This expression for temperature τ clearly generalizes our earlier result (S54) for the special case $p'_I = p'_E = 0$.

We recall that the meaningful range of λ is $0 \leq \lambda < 1/2$, so the temperature τ in Eq. (S82) is non-negative.

S4 Model: no restrictions on error rates

As discussed in Section S3.2 above, we have been unable to find a closed-form solution for the stationary-state feature frequency ρ, isogloss density σ and temperature τ for the fully general model, i.e. when all of the parameters p_I, p_E, p'_I, p'_E and q are in free variation. The root of the problem lies in the fact that the equations for the first and second moments of the distribution of features do not close, when $p'_I \neq p'_E$. In such cases there is either a tendency towards introducing a feature in the horizontal process, or towards losing it, amounting effectively to selection for or against the feature. Truncation schemes would be required to obtain approximations for the stationary values for ρ and σ. Interestingly very similar issues are known in the population genetic literature, in the presence of selection (*28*). We have not attempted to explore approximations for ρ and σ based on truncations of the resulting hierarchy of equations. However, the following ansatz is reasonable, and, as we show below, sufficiently accurate for our purposes.

In the model with $p'_I = p'_E$, the stationary-state feature frequency reads [see Eq. (S67)]

$$\rho = \frac{(1-q)p_I + q\lambda}{(1-q)(p_I + p_E) + 2q\lambda}, \tag{S83}$$

with $\lambda = p'_I = p'_E$, so $2\lambda = p'_I + p'_E$. Since ρ is by definition the frequency of up-spins, the following generalization of (S83) suggests itself:

$$\rho = \frac{(1-q)p_I + qp'_I}{(1-q)(p_I+p_E) + q(p'_I + p'_E)}. \tag{S84}$$

Secondly, when $p'_I = p'_E$, the stationary-state temperature reads [see Eq. (S82)]

$$\tau = \frac{(1-q)(p_I+p_E) + 2q\lambda}{q(1-2\lambda)}. \tag{S85}$$

Substituting again $p'_I + p'_E$ for 2λ yields the following ansatz for the general case $p'_I \neq p'_E$:

$$\tau = \frac{(1-q)(p_I+p_E) + q(p'_I + p'_E)}{q(1-(p'_I + p'_E))}. \tag{S86}$$

Finally, from the arguments in Section S3.3, we hypothesize the following form for the stationary-state isogloss density for the fully general model:

$$\sigma = 2H(\tau)\rho(1-\rho), \tag{S87}$$

where

$$H(\tau) = \frac{\pi(1+\tau)}{2K\left(\frac{1}{1+\tau}\right)} - \tau \tag{S88}$$

and τ is taken from Eq. (S86).

To corroborate these hypotheses, we ran a simulation of the model on a lattice of 50×50 sites and 100 features, with randomly drawn values for the parameters p_I, p_E, p'_I, p'_E and q for each feature, measured the empirical values of ρ, σ and τ at the end of each simulation, and correlated these against the above theoretical predictions. Each simulation was run for 25,000,000 iterations, so each feature underwent either a vertical or a horizontal event in each lattice site approximately 100 times during the simulation. The correlations are visualized in Fig. S1. The fact that all points lie close to the diagonal suggests that Equations (S84), (S85), (S86), even though not rigorously derived from theory, do faithfully describe these quantities at the stationary distribution. The Spearman correlation coefficients are $r_S = 0.994$ ($p < 0.001$) for ρ, $r_S = 0.985$ ($p < 0.001$) for σ, and $r_S = 0.931$ ($p < 0.001$) for τ.

S5 Robustness of method

To establish that our method for estimating empirical temperatures is robust against minor variations in the data it is fed, we have repeated the analysis by applying it to disjoint subsets of the WALS (*32*) dataset and by applying different spatial scales in the computation of isogloss densities, on whose values our empirical temperature estimates are based. We have also run the model on a neighbourhood graph inferred from the WALS atlas (instead of a regular lattice, as in the analytical solution), to assess to what extent the less regular real-life distribution of languages over physical space might invalidate the analytical regular-lattice idealization. Finally, in order to assess whether the irregular spatial distributions of genealogical units (language families and genera, rather than individual languages) might skew isogloss densities in unpredictable and non-universal ways, we have repeated the analysis with the restriction that nearest neighbour languages are always drawn from within the language family the focal language belongs to. The results of these extensions, which support our original idealized lattice model and the choice to consider only a small number of nearest neighbours for each language in the calculation of isogloss densities, are reported in the following subsections.

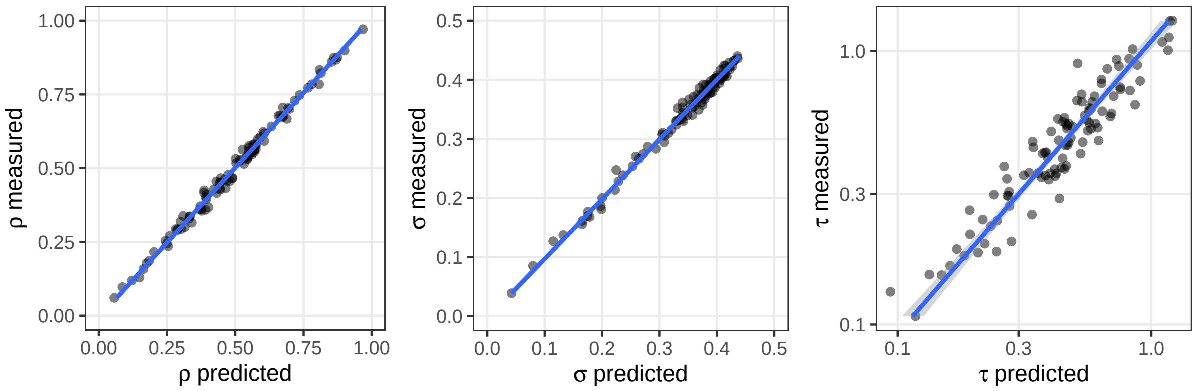

Figure S1. Correlations between quantities predicted by theory [Eqs. (S84), (S86) and (S87)] and measured from numerical simulations (ρ: feature frequency, σ: isogloss density, τ: temperature). One hundred independent features on a 50×50 lattice at 25,000,000 iterations.

S5.1 The two hemispheres test

Our main analysis relies on a bootstrap scheme to arrive at estimates of temperature τ. To make sure that τ is actually catching a signal rather than the particularities of an arbitrary sample of the world's languages, the procedure is repeated 1,000 times and the median temperatures and bootstrap distributions reported in the main paper. The question remains, however, to what extent temperature estimates from one sample of languages correlate with estimates from another such sample—a robust method should arrive at a similar ranking of features with respect to temperature τ regardless of which set of languages is considered, provided that that set is large enough. To answer this, we follow (*9*) by first partitioning the world into two subsets which are both genealogically and areally independent to a great degree: Old World (OW), consisting of longitudes between 0° E and 180° E, and New World (NW), comprising latitudes between 0° W and 180° W. The analysis is now conducted for both subsets (OW and NW) separately, and the median τ estimates from the OW bootstrap correlated against the median τ estimates from the NW bootstrap. The Spearman correlation coefficient was found to be $r_S = 0.52$ ($p < 0.01$). This should be compared against the value $r_S = 0.51$, which is what has previously been reported for a similar two hemispheres comparison based on a phylogenetic–areal technique (*9*). This reasonably high (and statistically significant) positive correlation suggests that temperature (τ) is a measurable quantity whose value is to some extent independent of the specific composition of the language sample used in its calculation.

S5.2 Varying the spatial scale

In the main paper, as well as in the above two extensions, we have computed isogloss densities σ on the basis of considering the 10 nearest geographical neighbours of each language—that is, assuming a neighbourhood size of $k = 10$. The question arises whether considering either smaller or larger neighbourhoods would noticeably affect our temperature estimates. To assess this, we repeated the analysis for neighbourhood sizes varying from $k = 1$ to $k = 50$ and correlated the temperature estimates against those found with $k = 10$ in the original analysis. The values of the Spearman correlation coefficients are plotted in Fig. S2 (left); all correlations are statistically significant at $p < 0.001$. The high correlations indicate that our method is robust over a range of spatial scales. Fig. S2 (right) shows that as neighbourhood size increases, the method becomes increasingly non-local. There is thus little incentive to consider larger neighbourhoods. This,

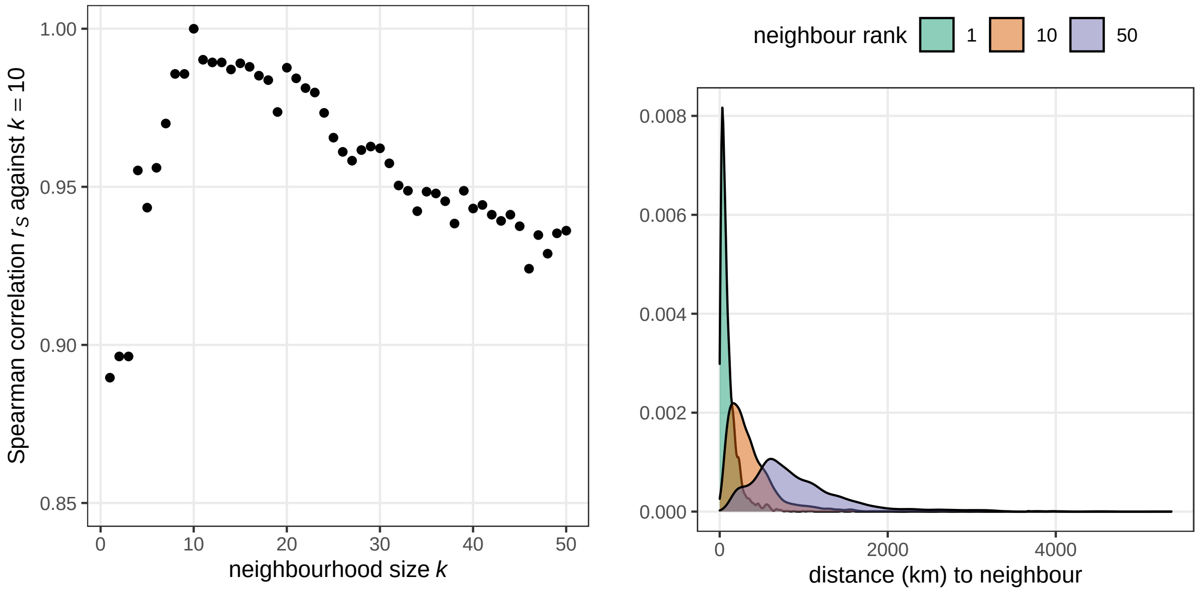

Figure S2. Left: Temperature estimates computed with varying neighbourhood sizes correlated against the temperature estimates of the main analysis (neighbourhood size $k = 10$). Right: Distribution (Gaussian kernel estimate) of distance to kth neighbour ($k = 1, 10, 50$).

moreover, does not exhaust the set of possible operationalizations of spatial neighbourhood. For example, the definition of isogloss density could incorporate geographical distance between languages such that the weight of any pair of languages decreases with increasing distance. Known physical barriers could also be taken into account.

S5.3 Spatial adjacency graph vs. regular lattice

The above analytical solution of the model assumes a regular isotropic infinite lattice. Empirically, languages are not distributed over physical space in this manner, so the question arises to what extent the spatial configuration of the mathematical model actually reflects reality. To assess this, we simulated the model on an adjacency graph inferred from the geographical coordinates of real languages extracted from WALS (altogether 2639 languages, disregarding the sign languages recorded in the atlas): each language was connected to its 10 nearest geographical neighbours. To simulate a variety of features of varying temperatures, 100 independent features were modelled, drawing the parameters p_I, p_E, p'_I, p'_E and q for each feature at random. Empirical values of feature frequency ρ, isogloss density σ and temperature τ were computed at the end of each simulation (after 25,000,000 iterations, so that each feature got updated in each language roughly 100 times). The empirical quantities were then correlated against what the theory predicts on an ideal lattice. The Spearman correlation coefficients are $r_S = 0.995$ ($p < 0.001$) for ρ, $r_S = 0.988$ ($p < 0.001$) for σ, and $r_S = 0.915$ ($p < 0.001$) for τ (Fig. S3).

S5.4 Potential baseline isogloss density bias resulting from the geographical distributions of languages belonging to a genealogical unit

It is possible to imagine a feature which is stable in the sense of having very low ingress and egress rates but being confined to a single genealogical unit (a language family or genus) whose languages happen to be scattered among languages from other genealogical units in which this feature is not present (cf. Fig. 2C in the main paper, imagining that the colours signify both

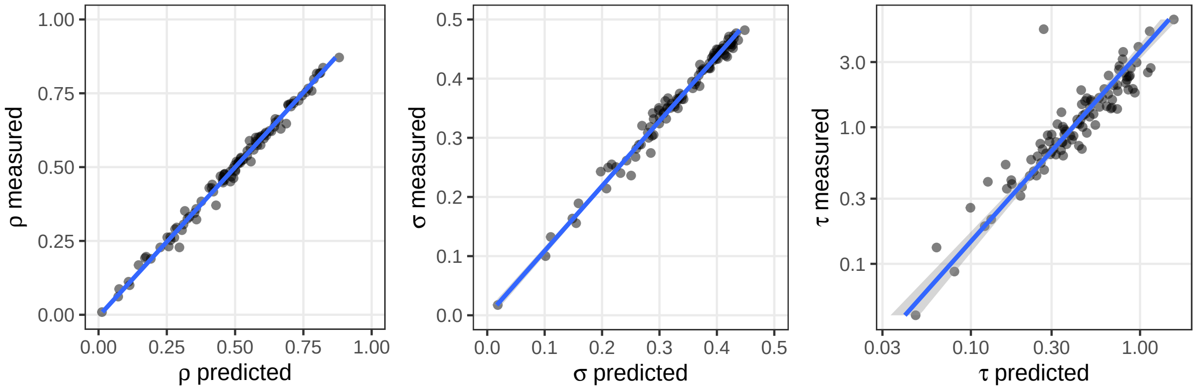

Figure S3. Correlations between quantities predicted by theory [Eqs. (S84), (S86) and (S87)] and measured from numerical simulations on an adjacency graph inferred from the WALS atlas (ρ: feature frequency, σ: isogloss density, τ: temperature). One hundred independent features at 25,000,000 iterations.

the presence of a feature as well as the genealogical unit, i.e. presence/absence of the feature precisely carves the genealogical boundary). In such a case, our method, which does not rely on genealogical information but only looks at the empirical spatial scattering, would conceivably report an intermediate (rather than low) temperature simply because of the areal scattering of the genealogical units, whereas a genealogical method would report maximal stability.

Certain features of our method render it relatively robust to such a scenario. Our main procedure estimates features' temperatures across languages belonging to several genealogical units; moreover, in the bootstrap set-up described in the main paper, the specific geographical composition of the language sample varies from one bootstrap run to the other. However, to make the point more quantitatively, we have implemented a variation of the original analysis, in which the nearest neighbour languages are always drawn from the same language family as the focal language, as specified in the genealogical information contained in WALS. On the assumption that the proposed scattered nature of the geospatial distributions of genealogical units causes a significant problem for our method, the temperature estimates from this genetic–areal variation of the method ought not to correlate, or at least not to correlate well, with those from our main analysis. This is, however, not the case. The Spearman correlation is 0.814, statistically significant at the $p < 0.001$ level.

S6 WALS feature levels

The following list gives the values of the WALS (*32*) features considered in this study; the italicized part after each value gives its value in our binarization (*present* for 'feature present', *absent* for 'feature absent' and *N/A* if the WALS value was excluded from the binarization as irrelevant). Languages attesting irrelevant values were excluded from the corresponding feature language sample for the purposes of calculating our statistics.

1. adpositions

- WALS feature: Person Marking on Adpositions (48A)
- Values:

  1: No adpositions (*absent*)
  2: No person marking (*present*)

3: Pronouns only (*present*)
4: Pronouns and nouns (*present*)

2. definite marker

– WALS feature: Definite Articles (37A)
– Values:

1: Definite word distinct from demonstrative (*present*)
2: Demonstrative word used as definite article (*present*)
3: Definite affix (*present*)
4: No definite, but indefinite article (*absent*)
5: No definite or indefinite article (*absent*)

3. front rounded vowels

– WALS feature: Front Rounded Vowels (11A)
– Values:

1: None (*absent*)
2: High and mid (*present*)
3: High only (*present*)
4: Mid only (*present*)

4. gender in independent personal pronouns

– WALS feature: Gender Distinctions in Independent Personal Pronouns (44A)
– Values:

1: In 3rd person + 1st and/or 2nd person (*present*)
2: 3rd person only, but also non-singular (*present*)
3: 3rd person singular only (*present*)
4: 1st or 2nd person but not 3rd (*present*)
5: 3rd person non-singular only (*present*)
6: No gender distinctions (*absent*)

5. glottalized consonants

– WALS feature: Glottalized Consonants (7A)
– Values:

1: No glottalized consonants (*absent*)
2: Ejectives only (*present*)
3: Implosives only (*present*)
4: Glottalized resonants only (*present*)
5: Ejectives and implosives (*present*)
6: Ejectives and glottalized resonants (*present*)
7: Implosives and glottalized resonants (*present*)
8: Ejectives, implosives, and glottalized resonants (*present*)

6. grammatical evidentials

– WALS feature: Semantic Distinctions of Evidentiality (77A)
– Values:

1: No grammatical evidentials (*absent*)
2: Indirect only (*present*)
3: Direct and indirect (*present*)

7. hand and arm identical

- WALS feature: Hand and Arm (129A)
- Values:

  1: Identical (*present*)
  2: Different (*absent*)

8. hand and finger(s) identical

- WALS feature: Finger and Hand (130A)
- Values:

  1: Identical (*present*)
  2: Different (*absent*)

9. indefinite marker

- WALS feature: Indefinite Articles (38A)
- Values:

  1: Indefinite word distinct from 'one' (*present*)
  2: Indefinite word same as 'one' (*present*)
  3: Indefinite affix (*present*)
  4: No indefinite, but definite article (*absent*)
  5: No definite or indefinite article (*absent*)

10. inflectional morphology

- WALS feature: Prefixing vs. Suffixing in Inflectional Morphology (26A)
- Values:

  1: Little affixation (*absent*)
  2: Strongly suffixing (*present*)
  3: Weakly suffixing (*present*)
  4: Equal prefixing and suffixing (*present*)
  5: Weakly prefixing (*present*)
  6: Strong prefixing (*present*)

11. inflectional optative

- WALS feature: The Optative (73A)
- Values:

  1: Inflectional optative present (*present*)
  2: Inflectional optative absent (*absent*)

12. lateral consonants

- WALS feature: Lateral Consonants (8A)
- Values:

  1: No laterals (*absent*)
  2: /l/, no obstruent laterals (*present*)
  3: Laterals, but no /l/, no obstruent laterals (*present*)
  4: /l/ and lateral obstruent (*present*)
  5: No /l/, but lateral obstruents (*present*)

13. morphological second-person imperative

- WALS feature: The Morphological Imperative (70A)
- Values:

  1: Second singular and second plural (*present*)
  2: Second singular (*present*)
  3: Second plural (*present*)
  4: Second person number-neutral (*present*)
  5: No second-person imperatives (*absent*)

14. order of adjective and noun is AdjN

- WALS feature: Order of Adjective and Noun (87A)
- Values:

  1: Adjective-Noun (*present*)
  2: Noun-Adjective (*absent*)
  3: No dominant order (*N/A*)
  4: Only internally-headed relative clauses (*N/A*)

15. order of degree word and adjective is DegAdj

- WALS feature: Order of Degree Word and Adjective (91A)
- Values:

  1: Degree word-Adjective (*present*)
  2: Adjective-Degree word (*absent*)
  3: No dominant order (*N/A*)

16. order of genitive and noun is GenN

- WALS feature: Order of Genitive and Noun (86A)
- Values:

  1: Genitive-Noun (*present*)
  2: Noun-Genitive (*absent*)
  3: No dominant order (*N/A*)

17. order of numeral and noun is NumN

- WALS feature: Order of Numeral and Noun (89A)
- Values:

  1: Numeral-Noun (*present*)

2: Noun-Numeral (*absent*)
3: No dominant order (*N/A*)
4: Numeral only modifies verb (*N/A*)

18. *order of object and verb is OV*

– WALS feature: Order of Object and Verb (83A)
– Values:

1: OV (*present*)
2: VO (*absent*)
3: No dominant order (*N/A*)

19. *order of subject and verb is SV*

– WALS feature: Order of Subject and Verb (82A)
– Values:

1: SV (*present*)
2: VS (*absent*)
3: No dominant order (*N/A*)

20. *ordinal numerals*

– WALS feature: Ordinal Numerals (53A)
– Values:

1: None (*absent*)
2: One, two, three (*present*)
3: First, two, three (*present*)
4: One-th, two-th, three-th (*present*)
5: First/one-th, two-th, three-th (*present*)
6: First, two-th, three-th (*present*)
7: First, second, three-th (*present*)
8: Various (*present*)

21. *passive construction*

– WALS feature: Passive Constructions (107A)
– Values:

1: Present (*present*)
2: Absent (*absent*)

22. *plural*

– WALS feature: Coding of Nominal Plurality (33A)
– Values:

1: Plural prefix (*present*)
2: Plural suffix (*present*)
3: Plural stem change (*present*)
4: Plural tone (*present*)
5: Plural complete reduplication (*present*)
6: Mixed morphological plural (*present*)

7: Plural word (*present*)
8: Plural clitic (*present*)
9: No plural (*absent*)

23. possessive affixes

- WALS feature: Position of Pronominal Possessive Affixes (57A)
- Values:

  1: Possessive prefixes (*present*)
  2: Possessive suffixes (*present*)
  3: Prefixes and suffixes (*present*)
  4: No possessive affixes (*absent*)

24. postverbal negative morpheme

- WALS feature: Postverbal Negative Morphemes (143F)
- Values:

  1: VNeg (*present*)
  2: [V-Neg] (*present*)
  3: VNeg&[V-Neg] (*present*)
  4: None (*absent*)

25. preverbal negative morpheme

- WALS feature: Preverbal Negative Morphemes (143E)
- Values:

  1: NegV (*present*)
  2: [Neg-V] (*present*)
  3: NegV&[Neg-V] (*present*)
  4: None (*absent*)

26. productive reduplication

- WALS feature: Reduplication (27A)
- Values:

  1: Productive full and partial reduplication (*present*)
  2: Full reduplication only (*present*)
  3: No productive reduplication (*absent*)

27. question particle

- WALS feature: Position of Polar Question Particles (92A)
- Values:

  1: Initial (*present*)
  2: Final (*present*)
  3: Second position (*present*)
  4: Other position (*present*)
  5: In either of two positions (*present*)
  6: No question particle (*absent*)

28. shared encoding of nominal and locational predication

- WALS feature: Nominal and Locational Predication (119A)
- Values:

  1: Different (*absent*)
  2: Identical (*present*)

29. tense-aspect inflection

- WALS feature: Position of Tense-Aspect Affixes (69A)
- Values:

  1: Tense-aspect prefixes (*present*)
  2: Tense-aspect suffixes (*present*)
  3: Tense-aspect tone (*present*)
  4: Mixed type (*present*)
  5: No tense-aspect inflection (*absent*)

30. tone

- WALS feature: Tone (13A)
- Values:

  1: No tones (*absent*)
  2: Simple tone system (*present*)
  3: Complex tone system (*present*)

31. uvular consonants

- WALS feature: Uvular Consonants (6A)
- Values:

  1: None (*absent*)
  2: Uvular stops only (*present*)
  3: Uvular continuants only (*present*)
  4: Uvular stops and continuants (*present*)

32. velar nasal

- WALS feature: The Velar Nasal (9A)
- Values:

  1: Initial velar nasal (*present*)
  2: No initial velar nasal (*present*)
  3: No velar nasal (*absent*)

33. verbal person marking

- WALS feature: Alignment of Verbal Person Marking (100A)
- Values:

  1: Neutral (*absent*)
  2: Accusative (*present*)
  3: Ergative (*present*)
  4: Active (*present*)

5: Hierarchical (*present*)
6: Split (*present*)

34. voicing contrast

- WALS feature: Voicing in Plosives and Fricatives (4A)
- Values:

  1: No voicing contrast (*absent*)
  2: In plosives alone (*present*)
  3: In fricatives alone (*present*)
  4: In both plosives and fricatives (*present*)

35. zero copula for predicate nominals

- WALS feature: Zero Copula for Predicate Nominals (120A)
- Values:

  1: Impossible (*absent*)
  2: Possible (*present*)

REFERENCES AND NOTES

1. D. Bickerton, Language evolution: A brief guide for linguists. *Lingua* **117**, 510–526 (2007).
2. M. Pagel, Human language as a culturally transmitted replicator. *Nat. Rev. Genet.* **10**, 405–415 (2009).
3. W. Labov, Transmission and diffusion. *Language* **83**, 344–387 (2007).
4. R. Jain, M. C. Rivera, J. A. Lake, Horizontal gene transfer among genomes: The complexity hypothesis. *Proc. Natl. Acad. Sci. U.S.A.* **96**, 3801–3806 (1999).
5. M. G. Newberry, C. A. Ahern, R. Clark, J. B. Plotkin, Detecting evolutionary forces in language change. *Nature* **551**, 223–226 (2017).
6. J. Nichols, *Linguistic Diversity in Space and Time* (University of Chicago Press, 1992).
7. E. Maslova, Dinamika tipologičeskix raspredelenij i stabil'nost' jazykovyx tipov. *Voprosy jazykoznanija* **5**, 3–16 (2004).
8. M. Parkvall, Which parts of language are the most stable? *STUF - Language Typology and Universals* **61**, 234–250 (2008).
9. S. Wichmann, E. W. Holman, *Temporal Stability of Linguistic Typological Features* (LINCOM Europa, 2009).
10. S. J. Greenhill, Q. D. Atkinson, A. Meade, R. D. Gray, The shape and tempo of language evolution. *Proc. R. Soc. B* **277**, 2443–2450 (2010).
11. D. Dediu, A Bayesian phylogenetic approach to estimating the stability of linguistic features and the genetic biasing of tone. *Proc. R. Soc. B* **278**, 474–479 (2011).
12. D. Dediu, M. Cysouw, Some structural aspects of language are more stable than others: A comparison of seven methods. *PLOS ONE* **8**, e55009 (2013).
13. S. Wichmann, in *The Routledge Handbook of Historical Linguistics*, C. Bowern, B. Evans, Eds. (Routledge, 2015), pp. 212–224.
14. S. J. Greenhill, C.-H. Wu, X. Hua, M. Dunn, S. C. Levinson, R. D. Gray, Evolutionary dynamics of language systems. *Proc. Natl. Acad. Sci. U.S.A.* **114**, E8822–E8829 (2017).
15. Y. Murawaki, K. Yamauchi, A statistical model for the joint inference of vertical stability and horizontal diffusibility of typological features. *J. Lang. Evol.* **3**, 13–25 (2018).
16. S. G. Thomason, T. Kaufman, *Language Contact, Creolization, and Genetic Linguistics* (University of California Press, 1988).
17. F. Gardani, P. Arkadiev, N. Amiridze, Eds., *Borrowed Morphology* (De Gruyter, 2014).

18. C. Bentz, B. Winter, Languages with more second language learners tend to lose nominal case. *Lang. Dyn. Chang.* **3**, 1–27 (2013).
19. J. Hey, C. A. Machado, The study of structured populations—New hope for a difficult and divided science. *Nat. Rev. Genet.* **4**, 535–543 (2003).
20. J. H. Greenberg, in *Universals of Human Language* , J. H. Greenberg, Ed. (Stanford Univ. Press, 1978), vol. 1, pp. 61–91.
21. C. Castellano, S. Fortunato, V. Loreto, Statistical physics of social dynamics. *Rev. Mod. Phys.* **81**, 591–646 (2009).
22. M. Kimura, G. H. Weiss, The stepping stone model of population structure and the decrease of genetic correlation with distance. *Genetics* **49**, 561–576 (1964).
23. P. Clifford, A. Sudbury, A model for spatial conflict. *Biometrika* **60**, 581–588 (1973).
24. T. M. Liggett, Stochastic models of interacting systems. *Ann. Probab.* **25**, 1–29 (1997).
25. B. L. Granovsky, N. Madra, The noisy voter model. *Stoch. Process. Their Appl.* **55**, 23–43 (1995).
26. M. J. de Oliveira, Linear Glauber model. *Phys. Rev. E* **67**, 066101 (2003).
27. P. L. Krapivsky, S. Redner, E. Ben-Naim, *A Kinetic View of Statistical Physics* (Cambridge Univ. Press, 2010).
28. K. S. Korolev, M. Avlund, O. Hallatschek, D. R. Nelson, Genetic demixing and evolution in linear stepping stone models. *Rev. Mod. Phys.* **82**, 1691–1718 (2010).
29. J. Kingman, The coalescent. *Stoch. Process. Their Appl.* **13**, 235–248 (1982).
30. J. F. Wilkins, J. Wakeley, The coalescent in a continuous, finite, linear population. *Genetics* **161**, 873–888 (2002).
31. J. Wakeley, *Coalescent Theory: An Introduction* (Roberts and Company Publishers, 2009).
32. M. S. Dryer, M. Haspelmath, Eds., *WALS Online* (Max Planck Institute for Evolutionary Anthropology, 2013).
33. M. P. Lewis, G. F. Simons, C. D. Fennig, Eds., *Ethnologue: Languages of the World* (SIL International, 2016).
34. J. Harrington, P. Hoole, F. Kleber, U. Reubold, The physiological, acoustic, and perceptual basis of high back vowel fronting: Evidence from German tense and lax vowels. *J. Phon.* **39**, 121–131 (2011).

35. G. K. Iverson, J. C. Salmons, in *Motives for Language Change*, R. Hickey, Ed. (Cambridge Univ. Press, 2003), pp. 199–212.

36. W. Strange, E. S. Levy, F. F. Law, II, Cross-language categorization of French and German vowels by naïve American listeners. *J. Acoust. Soc. Am.* **126**, 1461–1476 (2009).

37. B. L. Rochet, in *Speech Perception and Linguistic Experience: Issues in Cross-Language Research*, W. Strange, Ed. (York Press, 1995), pp. 379–410.

38. J. Janhunen, On the structure of Proto-Uralic. *Finnisch-Ugrische Forschungen* **44**, 23–42 (1982).

39. M. Erdal, *A Grammar of Old Turkic* (Brill, 2004).

40. J. H. Greenberg, in *Universals of Human Language* , J. H. Greenberg, Ed. (MIT Press, 1963), pp. 73–113.

41. R. Axelrod, The dissemination of culture: A model with local convergence and global polarization. *J. Confl. Resolut.* **41**, 203–226 (1997).

42. M. A. Beaumont, W. Zhang, D. J. Balding, Approximate Bayesian computation in population genetics. *Genetics* **162**, 2025–2035 (2002).

43. C. E. G. Amorim, R. Bisso-Machado, V. Ramallo, M. C. Bortolini, S. L. Bonatto, F. M. Salzano, T. Hünemeier, A Bayesian approach to genome/linguistic relationships in native South Americans. *PLOS ONE* **8**, e64099 (2013).

44. G. J. Baxter, R. A. Blythe, W. Croft, A. J. McKane, Utterance selection model of language change. *Phys. Rev. E* **73**, 046118 (2006).

45. J. Burridge, Spatial evolution of human dialects. *Phys. Rev. X* **7**, 031008 (2017).

46. S. Wichmann, Modeling language family expansions. *Diachronica* **34**, 79–101 (2017).

47. B. Comrie, M. S. Dryer, D. Gil, M. Haspelmath, *The World Atlas of Language Structures Online*, M. S. Dryer, M. Haspelmath, Eds. (Max Planck Institute for Evolutionary Anthropology, 2013).

48. T. Morita, Useful procedure for computing the lattice Green's function—Square, tetragonal, and bcc lattices. *J. Math. Phys.* **12**, 1744–1747 (1971).

49. R. C. Fernow, *Principles of Magnetostatics* (Cambridge Univ. Press, 2016).
